# Microbial Co-occurrence Relationships in the Human Microbiome

**DOI:** 10.1371/journal.pcbi.1002606

**Published:** 2012-07-12

**Authors:** Karoline Faust, J. Fah Sathirapongsasuti, Jacques Izard, Nicola Segata, Dirk Gevers, Jeroen Raes, Curtis Huttenhower

**Affiliations:** 1 Department of Structural Biology, VIB, Brussels, Belgium; 2 Department of Applied Biological Sciences (DBIT), Vrije Universiteit Brussel, Brussels, Belgium; 3 Department of Biostatistics, Harvard School of Public Health, Boston, Massachusetts, United States of America; 4 Department of Molecular Genetics, Forsyth Institute, Cambridge, Massachusetts, United States of America; 5 Department of Oral Medicine, Infection and Immunity, Harvard School of Dental Medicine, Boston, Massachusetts, United States of America; 6 Microbial Systems and Communities, Broad Institute of MIT and Harvard, Cambridge, Massachusetts, United States of America; The Centre for Research and Technology, Hellas, Greece

## Abstract

The healthy microbiota show remarkable variability within and among individuals. In addition to external exposures, ecological relationships (both oppositional and symbiotic) between microbial inhabitants are important contributors to this variation. It is thus of interest to assess what relationships might exist among microbes and determine their underlying reasons. The initial Human Microbiome Project (HMP) cohort, comprising 239 individuals and 18 different microbial habitats, provides an unprecedented resource to detect, catalog, and analyze such relationships. Here, we applied an ensemble method based on multiple similarity measures in combination with generalized boosted linear models (GBLMs) to taxonomic marker (16S rRNA gene) profiles of this cohort, resulting in a global network of 3,005 significant co-occurrence and co-exclusion relationships between 197 clades occurring throughout the human microbiome. This network revealed strong niche specialization, with most microbial associations occurring within body sites and a number of accompanying inter-body site relationships. Microbial communities within the oropharynx grouped into three distinct habitats, which themselves showed no direct influence on the composition of the gut microbiota. Conversely, niches such as the vagina demonstrated little to no decomposition into region-specific interactions. Diverse mechanisms underlay individual interactions, with some such as the co-exclusion of Porphyromonaceae family members and *Streptococcus* in the subgingival plaque supported by known biochemical dependencies. These differences varied among broad phylogenetic groups as well, with the Bacilli and Fusobacteria, for example, both enriched for exclusion of taxa from other clades. Comparing phylogenetic versus functional similarities among bacteria, we show that dominant commensal taxa (such as Prevotellaceae and *Bacteroides* in the gut) often compete, while potential pathogens (e.g. *Treponema* and *Prevotella* in the dental plaque) are more likely to co-occur in complementary niches. This approach thus serves to open new opportunities for future targeted mechanistic studies of the microbial ecology of the human microbiome.

## Author Summary

The human body is a complex ecosystem where microbes compete, and cooperate. These interactions can support health or promote disease, e.g. in dental plaque formation. The Human Microbiome Project collected and sequenced ca. 5,000 samples from 18 different body sites, including the airways, gut, skin, oral cavity and vagina. These data allowed the first assessment of significant patterns of co-presence and exclusion among human-associated bacteria. We combined sparse regression with an ensemble of similarity measures to predict microbial relationships within and between body sites. This captured known relationships in the dental plaque, vagina, and gut, and also predicted novel interactions involving members of under-characterized phyla such as TM7. We detected relationships necessary for plaque formation and differences in community composition among dominant members of the gut and vaginal microbiomes. Most relationships were strongly niche-specific, with only a few hub microorganisms forming links across multiple body areas. We also found that phylogenetic distance had a strong impact on the interaction type: closely related microorganisms co-occurred within the same niche, whereas most exclusive relationships occurred between more distantly related microorganisms. This establishes both the specific organisms and general principles by which microbial communities associated with healthy humans are assembled and maintained.

## Introduction

In nature, organisms rarely live in isolation, but instead coexist in complex ecologies with various symbiotic relationships [1]. As defined in macroecology, observed relationships between organisms span a wide range including win-win (mutualism), win-zero (commensalism), win-lose (parasitism, predation), zero-lose (amensalism), and lose-lose (competition) situations [2], [3], [4]. These interactions are also widespread in microbial communities, where microbes can exchange or compete for nutrients, signaling molecules, or immune evasion mechanisms [4], [5], [6]. While such ecological interactions have been recently studied in environmental microbial communities [7], [8], [9], [10], it is not yet clear what the range of normal interactions among human-associated microbes might be, nor how their occurrence throughout a microbial population may influence host health or disease [11].

Several previous studies have identified individual microbial interactions that are essential for community stability in the healthy commensal microbiota [12], [13], [14], [15], and many are further implicated in dysbioses and overgrowth of pathogens linked to disease [16]. Each human body site represents a unique microbial landscape or niche [17], [18], and relationships analogous to macroecological “checkerboard patterns” [3] of organismal co-occurrence have been observed due to competition and cooperation [5], [9], [19], [20]. For example, dental biofilm development is known to involve complex bacterial interactions with specific colonization patterns [21], [22], [23]. Likewise, disruption of relationships among the normal intestinal microbiota by overgrowth of competitive pathogenic species can lead to diseases, e.g. colonization of *Clostridium difficile* in the gut [24]. However, no complete catalog of normally occurring interactions in the human microbiome exists, and characterizing these co-occurrence and co-exclusion patterns across body sites would elucidate both their contributions to health and the basic biology of their ecological relationships. Thus, characterizing key microbial interactions of any ecological type within the human body would serve as an important first step for studying and understanding transitions among various healthy microbial states or into disease-linked imbalances.

As has been also been pointed out in macroecology, however, the analytical methodology needed to comprehensively detect such co-occurrence relationships is surprisingly complex [25]. Most existing studies employ simple measures such as Pearson's or Spearman's correlation to identify significant abundance relationships [13], [15], [26]. These methods are suboptimal when applied without modification to organismal relative abundances [27]. Since absolute microbial counts are not known and measurements depend on sampling and sequencing depth, an increase in one relative abundance must be accompanied by a compositional decrease in another, leading to spurious correlations among non-independent measurements [28]. In addition, sparse sequence counts can cause artefactual associations for low-abundance organisms with very few non-zero observations [27]. Conversely, association methods such as log-ratio based distances [28] that have been developed specifically for such compositional data are difficult to assign statistical significance, a vital consideration in high-dimensional microbial communities containing hundreds or thousands of taxa.

Here, we have addressed these issues to catalog a baseline of normal microbial interactions in the healthy human microbiome. The Human Microbiome Project (HMP) [29] sampled a disease-free adult population of 239 individuals, including 18 body habitats in five areas (oral, nasal, skin, gut, and urogenital), providing 5,026 microbial community compositions assessed using 16S rRNA gene taxonomic marker sequencing [29]. We have developed a suite of methods to characterize microbial co-occurrence and co-exclusion patterns throughout the healthy human microbiome while suppressing spurious correlations. Specifically, these were 1) an ensemble approach including multiple similarity and dissimilarity measures, and 2) a compendium of generalized boosted linear models (GBLMs) describing predictive relationships, both assessed nonparametrically for statistical significance while mitigating the effects of compositionality. Together, these methods provide a microbiome-wide network of associations both among individual microbes and between entire microbial clades.

Among the 726 taxa and 884 clades in the HMP data, we examined both intra-body site and inter-body site relationships as a single integrated microbial co-occurrence network. Each relationship represents co-occurrence/co-exclusion pattern between a pair of microbes within or between body sites among all subjects in the HMP (in contrast to studies within single subjects of microbial co-occurrences across biogeography, e.g. [30], [31]). This ecological network proved to contain few highly connected (hub) organisms and was, like most biological networks, scale-free. Co-occurrence patterns of the human microbiome were for the most part highly localized, with most relationships occurring within a body site or area, and there were proportionally few strong correspondences spanning even closely related body sites. Each pair of organisms was assessed for positive (e.g. cooperative) or negative (e.g. competitive) associations, and in many cases these patterns could be explained by comparing the organisms' phylogenetic versus functional similarities. In particular, taxa with close evolutionary relationships tended to positively associate at a few proximal body sites, while distantly related taxa with functional similarities tended to compete. The resulting network of microbial associations thus provides a starting point for further investigations of the ecological mechanisms underlying the establishment and maintenance of human microbiome structure.

## Results/Discussion

**Figure 1:**
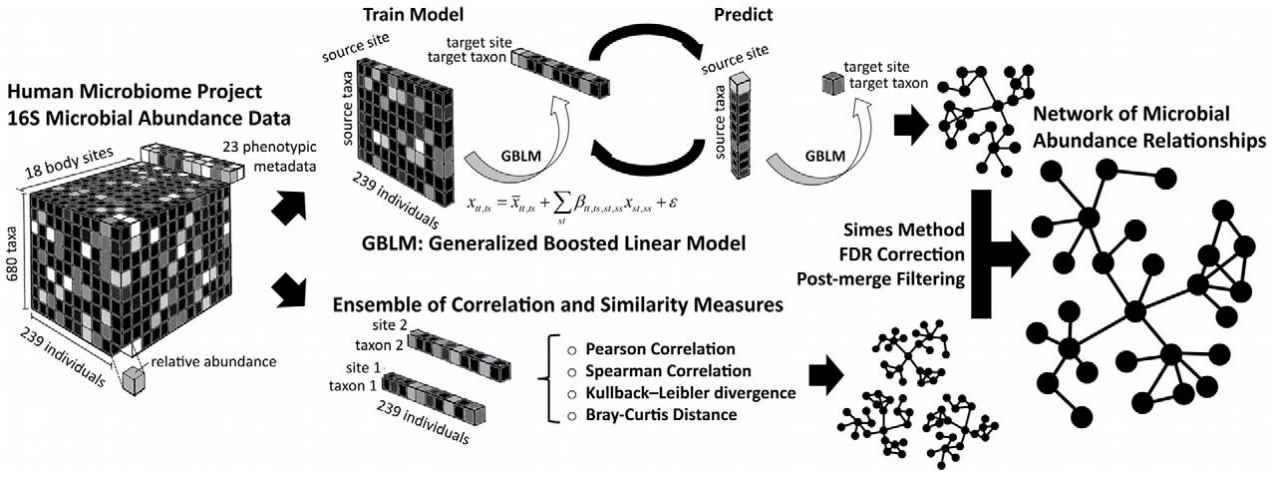
Methodology for characterizing microbial interactions using a compendium of similarity measures. 16S data from the Human Microbiome Project (HMP) were collected from 18 body sites in a cohort of 239 healthy subjects and assessed using 16S rRNA gene sequencing. We analyzed microbial co-occurrence and co-exclusion patterns in these data by developing two complementary approaches: a compendium of Generalized Boosted Linear Model (GBLMs) and an ensemble of similarity and dissimilarity measures. Each approach produced a network in which each node represented a microbial taxon within one body site, and each edge represented a significant association between microbial or whole clade abundances within or across body sites. The resulting association networks produced by each individual method were merged as p-values using Simes method, after which FDR correction was performed. Associations with FDR q-values>0.05, inconclusive directionality, or fewer than two supporting pieces of evidence were removed. This provided a single global microbial association network for taxa throughout the healthy commensal microbiota.

**Figure 2:**
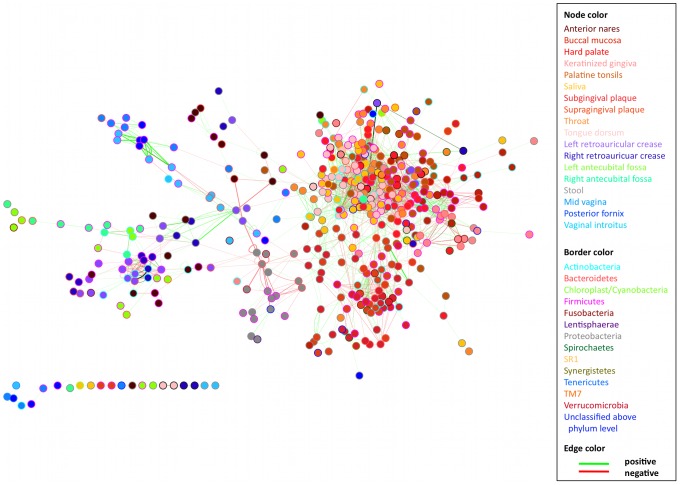
Significant co-occurrence and co-exclusion relationships among the abundances of clades in the human microbiome. A global microbial interaction network capturing 1,949 associations among 452 clades at or above the order level in the human microbiome, reduced for visualization from the complete network in Figure S1. Each node represents a bacterial order, summarizing one or more genus-level phylotypes and family-level taxonomic groups. These are colored by body site, and each edge represents a significant co-occurrence/co-exclusion relationship. Edge width is proportional to the significance of supporting evidence, and color indicates the sign of the association (red negative, green positive). Self-loops indicate associations among phylotypes within an order; for a full network of all phylotypes and clades, see Figure S1. A high degree of modularity is apparent within body areas (skin, urogenital tract, oral cavity, gut, and airways) and within individual body sites, with most communities forming distinct niches across which few microbial associations occur.

We inferred a microbiome-wide microbial interaction network by analyzing 5,026 samples from the Human Microbiome Project (HMP) comprising 18 body sites, 239 individuals recruited at two clinical centers, and 726 bacterial phylotypes detected by 16S rRNA gene sequencing (Table 1). Our study aimed to determine co-occurrence and co-exclusion relationships among the relative abundances of microbial taxa across all individuals, potentially indicative of their ecological relationships. We thus combined two complementary approaches, namely an ensemble of multiple similarity and dissimilarity measures (henceforth “ensemble approach”) and a compendium of generalized boosted linear models (GBLMs, henceforth “GBLM approach”). Both methods were applied to the HMP data to produce microbial interaction networks in which each node represented a microbial clade (taxon or group of taxa) connected by edges that were weighted by the significance of their association (positive or negative). Spurious correlations due to compositional structure of relative abundance data [27] were prevented by a novel bootstrap and re-normalization approach assessing the degree of association present beyond that expected by compositionality alone. We used Simes method followed by Benjamini-Hochberg-Yekutieli false discovery rate (FDR) correction to combine the resulting networks (Figure 1). A detailed final network is provided in Figure S1, with a comparison of all networks in Figure S7 and additional information in Methods. This provided a single global microbial interaction network capturing 3,005 associations among 197 phylotypes, spanning all available body sites from the human microbiome (Figure 2; Table S1).

**Table 1:** 16S rRNA gene sequencing data from the Human Microbiome Project used to assess microbial co-occurrence relationships in the human microbiome.

		Houston			St. Louis		
Body Area/Site	Total	Total	Female	Male	Total	Female	Male
***Oral***	*3022*	*2038*	*840*	*1198*	*984*	*456*	*528*
**Buccal mucosa**	340	228	92	136	112	53	59
**Hard palate**	334	221	90	131	113	53	60
**Keratinized gingival**	337	226	95	131	111	51	60
**Palatine Tonsils**	340	225	92	133	115	54	61
**Saliva**	309	227	94	133	82	35	47
**Subgingival plaque**	341	228	92	136	113	53	60
**Supragingival plaque**	349	232	97	135	117	55	62
**Throat**	321	219	92	127	102	46	56
**Tongue dorsum**	351	232	96	136	119	56	63
***Gut***	*351*	*228*	*94*	*134*	*123*	*58*	*65*
**Stool**	351	228	94	134	123	58	65
***Airways***	*282*	*190*	*82*	*108*	*92*	*37*	*55*
**Anterior nares**	282	190	82	108	92	37	55
***Skin***	*921*	*554*	*233*	*321*	*367*	*159*	*208*
**Left Antecubital fossa**	158	85	37	48	73	25	48
**Right Antecubital fossa**	160	83	33	50	77	34	43
**Left Retroauricular crease**	303	198	87	111	105	50	55
**Right Retroauricular crease**	300	188	76	112	112	50	62
***Urogenital***	*450*	*286*	*286*	*0*	*164*	*164*	*0*
**Mid vagina**	149	93	93	0	56	56	0
**Posterior fornix**	150	95	95	0	55	55	0
**Vaginal introitus**	151	98	98	0	53	53	0
***Total***	***5026***	***3296***	***2230***	***2324***	***1730***	***1292***	***1184***

We considered microbial associations in a total of 5,026 samples from the Human Microbiome Project (HMP) comprising 18 body sites in 239 individuals recruited at two clinical centers (Baylor College of Medicine, Houston, TX and Washington University at St. Louis, MO), which in total contained 726 reliably detectable bacterial phylotypes. For details of HMP samples and data processing, see [29].

### A global network of microbial co-occurrence and mutual exclusion within and among body site niches of the human microbiome

**Figure 3:**
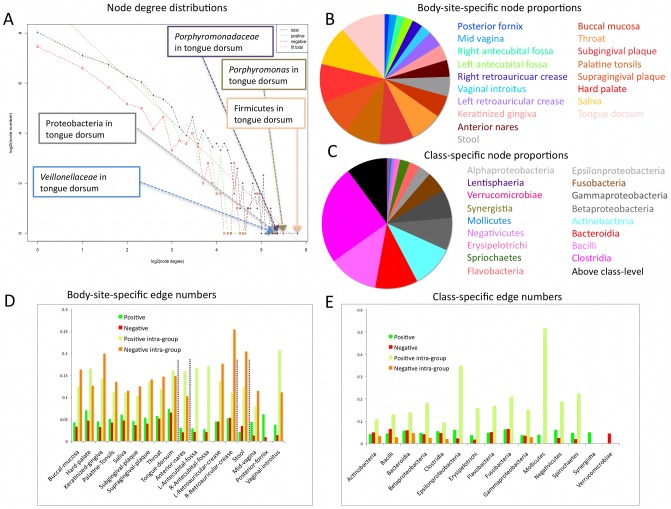
Global network properties summarizing key microbial hubs and interaction patterns. A) Node degree distributions of overall, co-occurrence, and co-exclusion associations in the human microbiome. This is well-fit by a power law with slope −1,7 (dotted red regression line, adjusted R^2^ = 0.9). Node degree indicates the number of links that connect a node to others in the network. Power law degree distributions, referred to as scale-free, mean that most nodes have only a few edges and are often connected by a few high-degree hub nodes. The top five most connected hubs as indicated in callouts, mainly signature oral taxa including *Porphyromonas* in the tongue dorsum. B) and C) Node proportions after division of the network into body sites (B) or classes (C). Both pie charts show that the composition of the network (in agreement with underlying data) is skewed towards the oral cavity (B) and its constituent Firmicutes (including Bacilli and Clostridia) (C). (B) further agrees with published measures of body sites' alpha diversity [84]. D) and E) Composition of relationships among microbes grouped according to body site (D) and taxonomic class (E). In E), the first two bars (green and red) include the fraction of all possible edges incident to at least one node representing a class or one of its members (root scaled for visualization). The second two bars (lime and orange) only include pairs of microbes that are members of the same class, again normalized as a fraction of total possible interactions and root scaled. The Bacilli, Bacteroidia, and Fusobacteria contain significantly more negatively associated microbes than expected by permutation testing (see Table S2), and classes overall are depleted for negative associations, indicating that members of the same class tend not to compete strongly with each other in these communities.

Global properties of the microbiome-wide network of microbial associations are summarized in Figures 2 and 3. A dominant characteristic of the network was its habitat-specific modularity. After grouping the 18 body sites into five broad areas (oral, skin, nasal, urogenital, and gut), the large majority of edges were found clustered within body areas (98.54%), and these clusters were sparsely connected through a minority of edges (1.46%). This is confirmed by the network's high modularity coefficient of 0.28 (as defined by [32]) and Markov clustering of the network (see Methods and Figure S2). It has long been observed that sites within the human microbiome are distinct in terms of microbial composition [33], and this proved to be true of microbial interactions as well: microbial relationships within each body area's community were largely unique (Table 2). The microstructure of interaction patterns - and thus in the underlying ecology - was different for different areas, however. For example, all vaginal sites within the urogenital area were interrelated in a single homogeneous community, whereas interactions within the oral cavity suggested microbial cross-talk among three distinct habitats [34]. This can be observed quantitatively based on the proportions of microbial interactions spanning body sites within each area, e.g. 69.57% among the vaginal sites and 53.19% among the oral sites, both exceeding the microbiome-wide baseline. The skin was further unique in that the large amount (57.65%) of its associations related microbes in corresponding left and right body sites (left and right antecubital fossae and retroauricular creases), reflecting consistent maintenance of bilateral symmetry in the skin microbiome.

**Table 2:** Summary statistics of microbial associations in the normal human microbiota.

Edges	# of edges	Percent
Within same body area	2961	98.54%
Within same body site	1409	(47.59%)
Among skin sites	196	6.52%
Between left and right skin sites	113	(57.65%)
Within the airways (anterior nares)	31	1.03%
Among oral sites	2598	86.46%
Between different oral sites	1382	(53.19%)
Within the gut	67	2.23%
Among vaginal sites	69	2.30%
Between different vaginal sites	48	(69.57%)
Total	3005	

Microbial co-occurrence and co-exclusion relationships summarized within the five major body areas and relationships spanning different body sites within these areas. Percentages are fractions of the total number of edges in the network, while percentages in parentheses represent fractions of edges within each body area.

We began decomposing the network by categorizing microbial associations within each body area into body-site-specific relationships of two types: cross-site and within-site interactions. On average, these two classes make up 53.11 and 46.89 percent of the total edges, respectively (Table 2). First focusing on cross-site associations, a majority (66.10%) of such relationships were co-occurrences between the same or taxonomically related clades in proximal or bilateral body sites. This reflects coordinated community structure among ecologically related niches, such as similar dental plaques, vaginal sites, and bilateral skin sites. Body sites specifically connected by many positive associations were either in direct contact (e.g. tongue and saliva), proximal (e.g. sub- and supragingival plaques), or similar in terms of environmental exposure (e.g. bilateral skin sites), thus providing mechanisms to support comparable microbiota and exhibiting high levels of microbial co-occurrence. This pattern held true for the minority (33.90%) co-exclusions as well, with many occurring between bilateral skin sites or within subgroups of the oral cavity [34]. This suggested that the first level of hierarchical co-occurrence structure in this network corresponded with groups of body sites representing distinct microbial habitats.

Conversely, within-site relationships showed a much more balanced ratio of microbial co-occurrence (48.26%) vs co-exclusion (51.74%) interactions. Many of the negative within-site relationships were associated with the abundant signature organisms characteristic of each body site [35], for example *Streptococcus* in the oral cavity and *Bacteroides* in the gut. The relative abundances of these signature taxa varied greatly among individuals, in some cases (e.g. *Bacteroides*) spanning from 1% to 97% within a body site across the HMP population. It is generally very difficult to determine from relative abundance measurements alone whether these negative associations represent true anti-correlation (e.g. one organism out-competing another) or overgrowth of one organism while the rest of the population remains unchanged (resulting in a negative correlation due to compositionality of these data). This problem has a long history in quantitative ecology [27], [28]. Our methods generally determine these relationships in the human microbiome to be stronger than what would be expected from compositionality alone (see Methods and Text S1), and the negative interactions detected here are thus likely biologically informative. This is supported by the fact that they are strongest in cases where distinct alternative dominant community members occurred among different individuals (e.g. Prevotellaceae vs. Lactobacillaceae in the vaginal area [36] or *Propionibacterium* vs. *Staphylococcus* on the skin [35], [37]). The increase in negative interactions within habitats is also in line with the fact that most competitive mechanisms require proximity or physical contact [38], whereas positive interactions are likely to also occur from microbiome-wide shared environmental exposures.

### Association properties globally and within body sites demonstrate the basic ecological organization of the human microbiota

We further assessed several other measures of network community structure. Globally speaking, the network followed a scale-free degree distribution typical of biological systems, meaning that most clades possessed few interactions but a few clades possessed many (Figure 3A [39]), The network had a low average path length of three (contrasted with six in randomized networks), meaning that short paths existed between most clades [40], and it possessed a low average per-node cluster coefficient (0.1) measuring the local density of connections. Together, these values indicate that the microbial association network is structured to be scale-free and thus robust to random disruption [39], with only sparse local multi-organism clusters. Since these data only describe phylotypes at approximately the genus level, it remains to be seen whether a greater degree of locally clustered functional associations emerges among Operational Taxonomic Units (OTUs), species, or strains within these phylotypes. As the cluster coefficient distribution was not well described by the inverse node degree distribution [41], the network possesses no strong hierarchical modularity despite its scale-freeness, in contrast to the strong habitat-centric modularity.

The diversity of microbial interactors (i.e. number of unique phylotypes) within each body site also proved to directly dictate its interaction density (Figure 3B). That is, communities with a greater number of different organisms had a proportionally greater number of positive and negative associations. Within these sites, the number of relationships scaled directly with the number of unique phylotypes (adjusted R^2^ of 0.75), the only body site with more interactions than expected for its diversity being the tongue dorsum (see also Table S2). This site also harbored the top-ranking hub phylotype (Firmicutes, see Figure 3A). In combination with the behavior of specific microbial hubs as discussed below, this might argue that most microbial taxa form strong metabolic or functional associations with adjacent taxa inhabiting the same body site habitat, allowing consortia to specialize within highly localized microbial niches [33].

When randomizing between rather than within body sites, no body site pairs possessed more cross-site associations than expected (with the slight exception of tongue dorsum), whereas most body sites were significantly enriched for within-site relationships (the only exceptions being posterior fornix, mid-vagina, and antecubital fossae, which tended toward too few phylotypes to reach significance; see Figure 3D and Table S2), again confirming the microbiome's habitat-driven modularity. When calculating network properties in a body-area-specific manner, we found that the overall average path length between nodes in the oral cavity, which contributes most of the samples, was much larger (∼3.4) than those of the other body areas (ranging from ∼1.1 to ∼2.0). In addition to supporting the aforementioned degree of inter-site habitat formation in the oral cavity, this intriguingly suggests that other body sites in which fewer samples are currently available (see Table 1) have not yet exhausted the detection of microbial relationships in the human microbiome. More samples and greater sequencing depth may further improve detection power.

### Key taxa including members of the Firmicutes act as network hubs coordinating many relationships throughout the microbiome

We next examined the associations of individual clades with respect to interaction degree, observing highly connected “hub” clades to be found within each body area. Two classes of hubs appeared in the association network: clades highly connected within one body site, and clades acting as “connectors” between multiple body sites. Hubs included both specific taxa (e.g. *Porphyromonas*, see Figure 3A, Table S3) and larger taxonomic groupings (e.g. the phylum Firmicutes). Within-site hubs were often, although not always, abundant signature taxa (detailed below), high-degree exceptions including *Atopobium* on the tongue (28 total associations, 16 within-site) and *Selenomonas* on both tooth plaques (20 total/19 within and 7 total/3 within for supra- and subgingival, respectively). The latter provides a striking example of the niche-specificity of these low-abundance within-site interactors, as *Selenomonas* averages only 1.1% and 1.2% of the sub- and supragingival plaque communities, respectively, but associates preferentially (20 of 27, 74%) with members of the greater oxygen availability supragingival community. The clade's detection as a within-site hub thus corresponds with the ecology that might be expected of an organism known to be oxygen-sensitive, fastidious, and grown best in co-culture [42].

Between-site hubs typically operated among body sites within the same area as described above, with two of the five most connected hub clades in the network falling into this connector category linking multiple body sites, Firmicutes and Proteobacteria on the tongue (see Figure 3A). The Firmicutes and *Porphyromonas* (phylum Bacteroidetes) hubs in the tongue also had the largest numbers of negative connections among all phylotypes, and all of these highly interactive clades centered on the tongue and spanned multiple related oral habitats. Signature clades such as the Firmicutes are of course highly functionally diverse, and this network suggests that the few abundant members in any one habitat [35] might instead serve as “information processors” throughout a body area. In contrast to the low-abundance within-site hubs, this would allow them to provide baseline functionality complemented by distinct, less abundant clades with which they co-occur within differing body site habitats.

Correspondingly, Firmicutes and other inter-site hub nodes showed a higher connectivity than the clades with highest intra-site degree (e.g. Bacteroidales in the subgingival plaque). Such clades with unusually frequent inter-site associations are thus outliers relative to the network's overall habitat-specific trend and suggest that inter-site hubs are particularly critical for associating similar sites within the same body area. In the oropharynx, for example, *Streptococcus* spp. with a modest degree of functional variation might be present throughout the habitat, interacting with distinct, more specialized clades within each body site [13]. Almost all such high-connectivity hubs occurred among oral sites (e.g. *Porphyromonas*, *Streptococcus*, *Veillonella*, and others), the first notable exception being the *Propionibacterium* hub on skin sites (left and right retroauricular crease). All of these follow the same pattern, however, in which abundant phylotypes likely possessing within-clade functional diversity are distributed among related habitats within each individual.

### Marked differences in ecological behavior between phylogenetic clades

We additionally examined the phylogenetic rather than biogeographical distribution of these associations, testing whether clades tended to support more phylogenetically related (within-clade) or diverse (between-clade) interactions. We first investigated purely quantitative degree distributions by summarizing clades at the class level. Associations were summarized as the fraction of all possible interactions that were observed to occur, separated into positive and negative bins (Figure 3E). In addition, clade-specific over-representation of these bins was tested for significance by randomization (see Methods and Table S2). The only classes that showed significantly more negative (and, simultaneously, cross-clade) associations than expected were the Bacteroidia, Bacilli, and Fusobacteria. Most of the common classes in the human microbiome had more intra-clade edges than expected by chance (Actinobacteria, Bacilli, Bacteroidia, Betaproteobacteria, Clostridia, Epsilonproteobacteria, Fusobacteria, Gammaproteobacteria, Mollicutes, and Spirochaetes), most of which also have high cluster coefficients (Figure S3). Taken together with the biogeographical interactions assessed above, the enrichment for within-class associations likely indicates a phylogenetic aspect of the same behavior. Specifically, if one member of such a class is abundant in one body site within an individual, it (or closely related class members) also tends to be enriched in related body sites.

**Figure 4:**
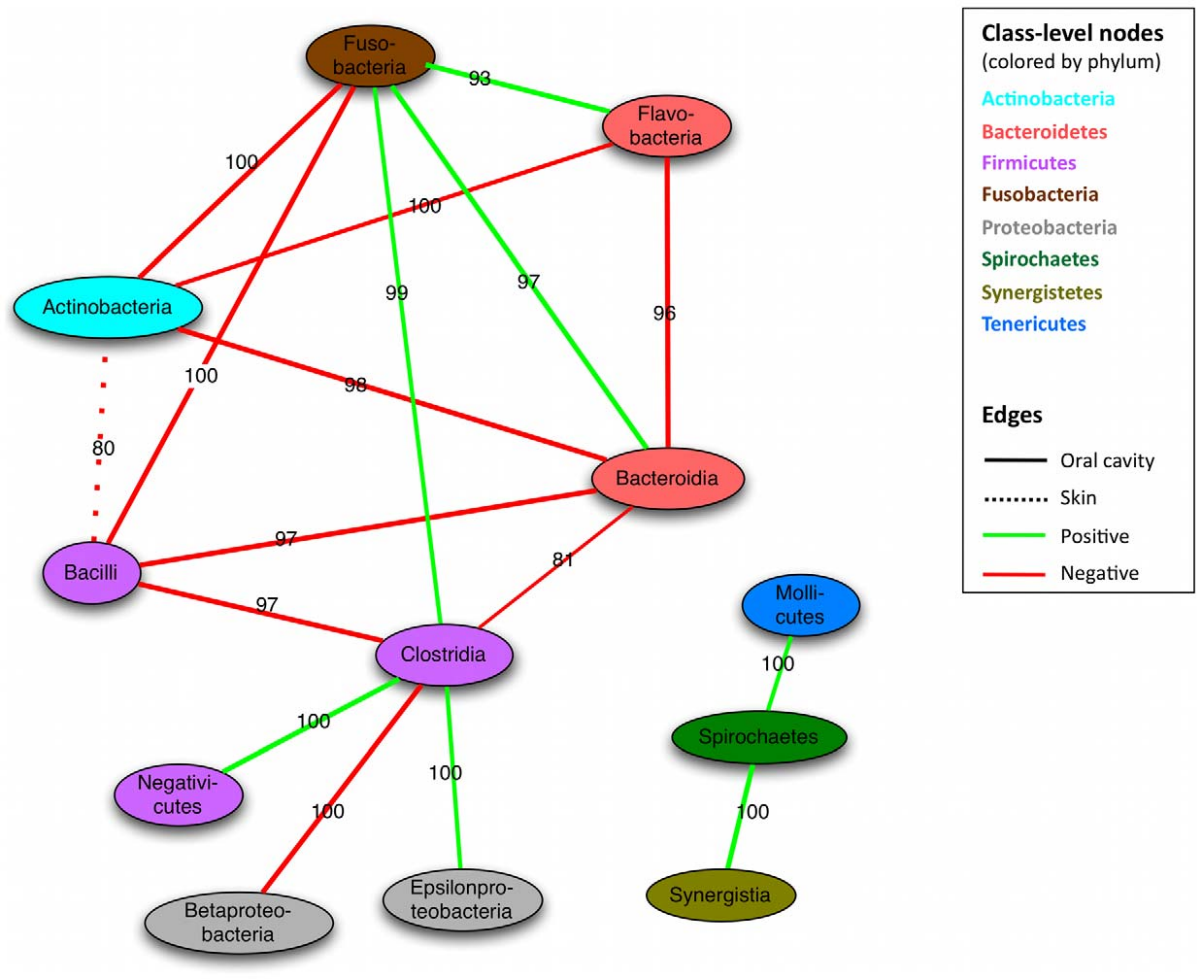
Co-occurrence of microbial clades within and among body areas. Nodes represent microbial classes colored by phylum, with edges summarizing aspects of their interactions over all body sites. Classes are linked when the number of edges between them is significantly larger than expected (randomization p<0.05, see Methods). Edge type (solid or dashed) indicates the body area contributing the most edges to the total interactions between two classes, with the label specifying the percentage contributed by this dominant body area. For instance, 80% of the edges between Bacilli and Actinobacteria come from skin sites. Green indicates co-occurrence, red exclusion. Most inter-class interactions occur in the mouth, with the Actinobacteria and Bacilli forming negative hubs.

We next considered relationships between class-level clades throughout the microbiome, summarized in Figure 4. Surprisingly, the Actinobacteria and Bacilli form only co-exclusion relationships with other classes, most strongly with Bacteroidia and Fusobacteria, and primarily within the oral cavity. These clades (which include the extremely abundant streptococci) might thus be largely self-sufficient in the functional diversity needed to maintain an oral community, excluding other clades when appropriately supported by e.g. environmental factors. Although a few classes were linked by positive as well as negative interactions (e.g. Clostridia and Bacteroidia), none of these reached significance on randomization. Classes connected by both positive and negative links might suggest either that the clades exhibit co-occurrence only in some environments or that some members of the two classes co-occur while others co-exclude. As the oral communities are both the most data-rich and the most alpha-diverse in the human microbiome [35], it is not surprising that most relationships are observed within and among them. For instance, 97% of the specific mutual exclusions between Bacilli and Bacteroidia members occur in oral sites, as do 81% of the members of the Clostridia and Bacteroidia. The second largest contribution to the latter exclusion (∼18%) comes from the gut, reflecting the frequently discussed Bacteroides/Firmicutes ratio observed in Western populations [15], [43], and similar tradeoffs (with few positive associations) were observed in other habitats such as the skin (e.g. *Staphylococcus* in the Bacilli and *Propionibacterium* in the Actinobacteria [37]).

Co-exclusions such as these have previously been observed in the human microbiota to induce distinct alternative community configurations, which may differ across persons [15], [36] as well as time points (e.g. early and late colonizers in community establishment or repopulation after disturbance). Although our methodology does not explicitly describe alternative community configurations, co-occurrence networks can in some cases capture them as extreme exclusion relationships between key microbial taxa. For instance, Ravel et. al reported five different vaginal communities in an independent cohort of healthy women, four dominated by Lactobacilli and the fifth diverse and featuring members of the Actinobacteria, Clostridia, Bacteroidia, and other classes. These alternative configurations occur as mutual exclusions in our genus-level phylotypes between *Lactobacillus* and members of this fifth diverse community (particularly anaerobes such as Anaerococcus and the Prevotellaceae). Furthermore, we see a strong negative correlation in stool samples between *Bacteroides* and members of the gut community, including the Ruminococcaceae and other Firmicutes. In other body sites, the clade relationship network (Figure 4) features a negative interaction between Bacilli and Bacteroidia classes that mostly occurs in the oral cavity, and oral Porphyromonas (a member of the Bacteroidia) is among the most highly connected negative hubs. *Porphyromonas* is abundant (avg. 3.3% s.d. 3.9%) in oral habitats but not in most cases the dominant clade; the clade also includes potential oral pathogens [44], and this may be one of the more striking examples of functional competition and co-exclusion occurring with a specific clade among several oral communities.

### Microbial relationships within digestive tract niches including *Fusobacterium* and *Prevotella* support known microbiology

**Figure 5:**
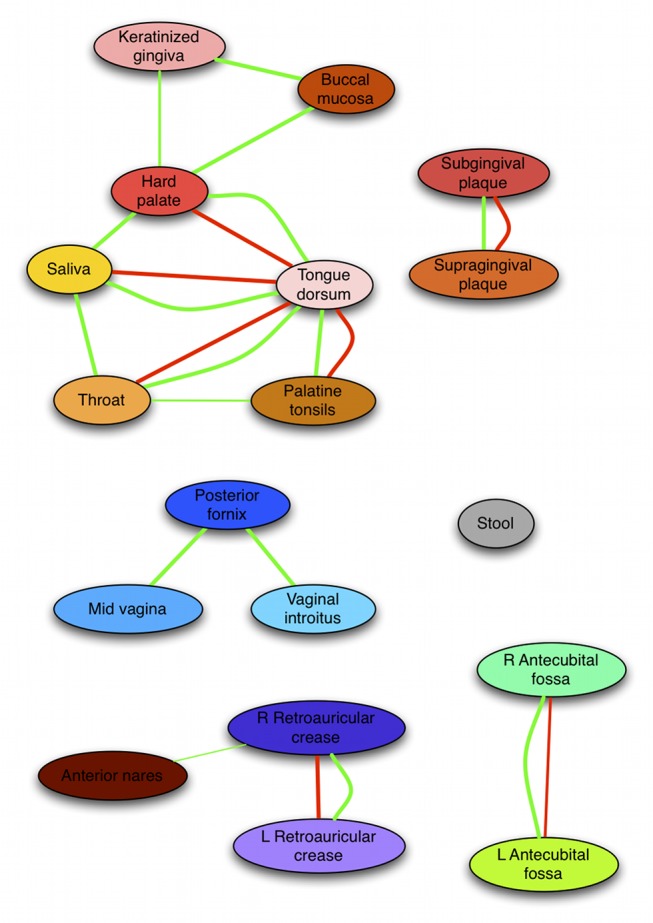
Related microbial niches as determined by associations spanning habitats at multiple human body sites. Each node represents a body site, with edge width indicating significant cross-site correlations (randomization p<0.05, see Methods). Green edges show co-occurrence, red co-exclusion. Skin, vaginal, oral soft tissue, and tooth plaque moieties are apparent, with the gut and airways notably lacking significant interactions with other available body site niches. However, most relationships between microbial relative abundances occur specifically within, rather than between, individual body sites.

The digestive tract is home to one of the most diverse and densely populated microbial communities in the human body [11]. Oral sites made up half of the body sites surveyed here, as well as exhibiting the greatest within-subject microbial diversity [35]. Correspondingly, associations between microbes within and among oral sites likewise comprised the majority (86.46%) of all edges in our co-occurrence network, also forming its largest connected component. This consisted of two clusters of organisms from the mouth soft tissues (gingiva, mucosa, and palate) and distal areas (tongue, throat, tonsils, and saliva); the oral hard surfaces (sub- and supra-gingival plaques) formed an additional isolated habitat that showed significantly fewer microbial associations with the remainder of the oral cavity (Figure 5). A complementary analysis of the HMP microbiomes has revealed evidence of three sub-habitats within the oral cavity based on overall similarity of their microbial communities [34], and these results demonstrate that the shared community structures of these habitats were to a lesser degree recapitulated in terms of specific microbial associations (see Figure 5 below).

Although the current study is associative and does not by itself establish causative mechanisms of interaction for these microbial associations, many that we detect in the oral cavity in particular are supportd by known metabolic or biochemical interactions. For instance, in the context of cell to cell interaction, *Fusobacterium* species are known to be bridging organisms in the development of oral biofilms by co-aggregation through physical contact [45]. This bridging occurs during biofilm maturation, allowing a more complex use of resources including sugars (the predominant carbon source for early colonizers) and proteins (used by late colonizers). In the hard palate, for example, positive associations were found between *Fusobacterium* and *Capnocytophaga*, *Peptosptreptococcus*, and *Porphyromonas*, which are in agreement with previously published cell-to-cell interactions [46], [47], and these predictions additionally implicate *Leptotrichia* and *Parvimonas*. Dental plaque associations included *Parvimonas*, *Prevotella*, and *Treponema*, also in agreement with existing evidence [48]. However, those previously published aggregations are strain specific and, this study may be observing broader effects than the direct cell-cell contact preferences in previously described associations.

Conversely, metabolic shifts may explain negative associations detected between other co-habiting microbes, e.g. *Tannerella* and *Streptococcus* in the subgingival plaque. The anaerobic *Tannerella* requires a much lower pO_2_ than *Streptococcus* and is proteolytic, while *Streptococcus* is a saccharolytic colonizer of the tooth surface that uses sugars as its primary source of carbon and is oxygen tolerant [49], [50]. This continuous nutritional, metabolite (e.g. hydrogen peroxide), and oxygen gradient between the supragingval and the subgingival biofilms, along with differential exposure to host factors in saliva, is reflected through the gradual drop of the abundance of *Tannerella* as the streptococci increase (Figure S4). A similar example can be found in the *Prevotella* and species from the Flavobacteriaceae (represented here by *Capnocytophaga*; mean abundance 1.68±2.76%) in the tonsils. Less exposed surfaces of tonsillar crypts offer an anaerobic micro-environment favoring species like *Prevotella*, while other areas support the growth of carbon dioxide-dependant *Capnocytophaga*, a tradeoff that we detect here as a specific negative association.

Phyla such as the TM7 and Synergistetes have only recently been characterized at the genetic level in the oral cavity [51], [52], and little is yet known about their roles in this microbial ecosystem. We identified a number of novel co-occurrences between members of these under-characterized phyla, including a positive association between members of the TM7 phylum (mean abundance 0.62±1.14%) and *Moryella* genus members (mean abundance 0.29±0.47%) in the tongue dorsum and a positive relationship between members of the Synergistetes phylum and *Treponema* in the subgingival biofilm. Since limited data on metabolic byproducts or requirements for these clades in the oral community are available, these newly identified putative interactors provide specific hypothesis for follow-up studies (e.g. by co-culture experiments).

The degree to which microbial shedding from the oral cavity along the digestive tract might seed the distal commensal gut microbiota is as yet unclear [53]. We found few (7) relationships between organisms in the two areas meeting our significance criteria, none of which were consistently supported by a majority of available data (Figure S5), suggesting no such direct microbial seeding within our level of detection in the healthy adult microbiome. Interactions detected within the gut itself consisted primarily of negative associations between *Bacteriodes* and *Clostridia*, especially members of the Ruminococcaceae family. These negative relationships reflect the tradeoff between *Bacteroides* (mean abundance 48.79±22.94%, range 1.47–97.14%) and Firmicutes (mean abundance 27.04±16.52%, range 1.49–91.78%), the two dominant gastrointestinal taxa and the subject of previous close study [15], [54]. While oral microbial transit is clearly important during founding of the microbiome in infancy and in extreme cases such as illness [55], [56], these data suggest that it occurs at low levels in the normal adult microbiome. In such hosts, the naturally dense microflora of the lower gut may serve to further exclude the few bacteria that survive gastrointestinal transit [53].

### Similarities among niches in the microbiome determined by microbial associations spanning body sites

It is common practice to group microbial communities by ecological similarity [33], [35], and we extended this analysis method by summarizing relationships among similar habitats based on microbial cross-talk (Figure 5). Specifically, we organized pairs of body sites by the frequency with which they demonstrated co-occurring (or excluding) microbes (see Methods). Overall, this network recapitulates similarities in community structure among these microbial habitats as assessed by beta-diversity [35], with the added information of which microbes might drive these similarities. Conversely, co-exclusions spanning multiple habitats might represent cases in which competitive relationships or differing responses to host environment might bridge multiple habitats. Stool microbes (representing the gut microbiota), as above, did not demonstrate any detectable associations with inhabitants of the mouth; the airways microbiota (nares) likewise associated minimally with other body sites, although they were detectably structurally similar to the skin communities. The sub- and supra-gingival plaques were distinct from other mouth sites, and the vaginal communities and skin were again all highly similar. The sparsity of this body site network again illustrates that phylotypes rarely participate in detectable ecological relationships spanning distal body site habitats.

### Functional and phylogenetic similarities among associated organisms suggest competitive and adaptive explanations for interactions

**Figure 6:**
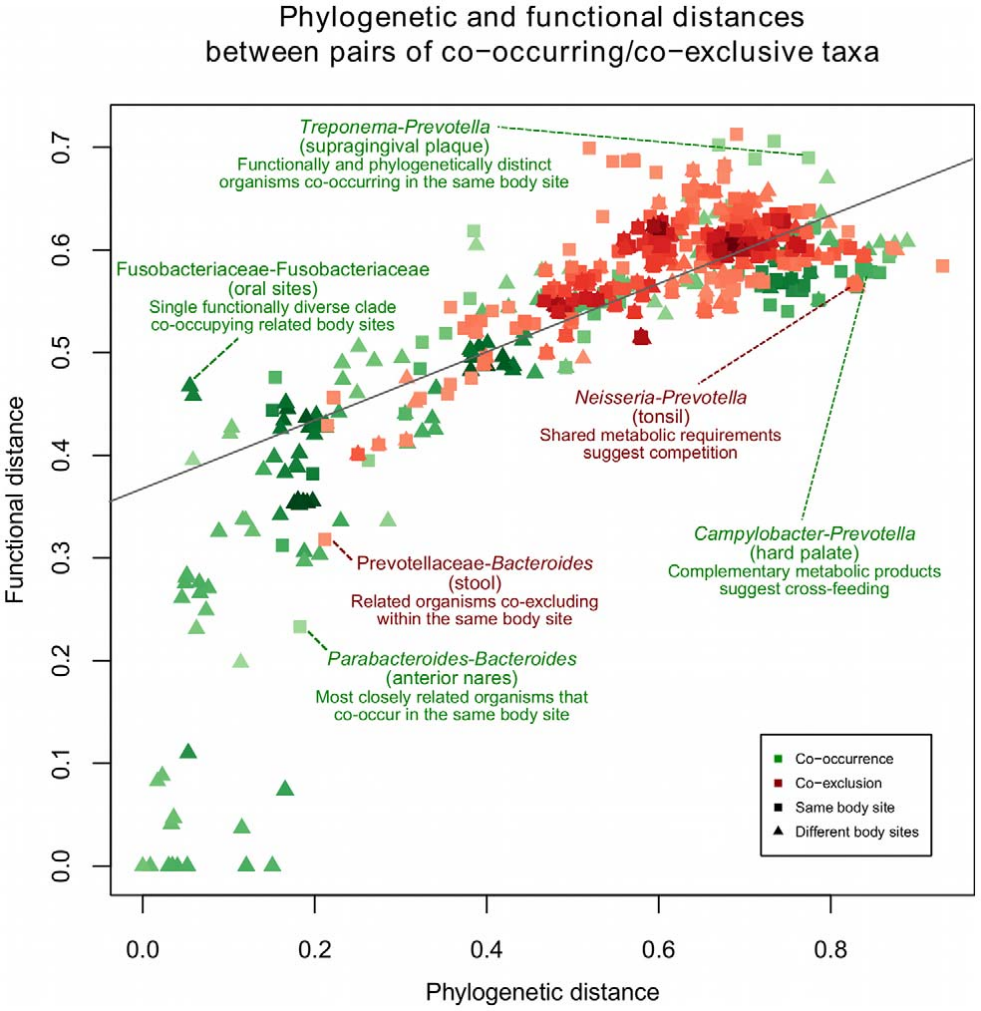
Functional and phylogenetic similarities between co-occurring microbes. Evolutionary (phylogenetic) distances among microbial clades were compared to the clades' functional potentials as defined by the Jaccard index of orthologous gene (COG) families shared between genomes (see Methods). Each point represents a pair of significantly associated microbes colored by direction of the association (green positive, red negative) and shaped by the type of relationship (triangle: between body sites, square: within site). Phylogenetic distances were inferred by FastTree [82] using species-level 16S sequences. Most interactions lie along the diagonal, reflecting the baseline correlation between these functional and evolutionary distances, with highly related clades co-occurring among related habitats (e.g. bilateral skin sites, proximal oral sites) in the lower left. Off-diagonal examples include potential competition among dominant gut signature taxa (e.g. Prevotellaceae/*Bacteroides*) and functional complementarity between distinct oral pathogens (e.g. *Treponema*/*Prevotella*).

We hypothesized based on previous findings in environmental communities [19] that patterns of microbial co-occurrence and exclusion might be explained by their evolutionary relatedness and functional similarity. For example, closely related microbes might compete for limited resources, while functionally complementary bacteria would exhibit mutualism. To test this hypothesis, we compared two genomic properties of all microbial clades appearing in our network, their phylogenetic similarity (i.e. evolutionary relatedness) and a “functional” similarity score based on counting shared orthologous gene families (i.e. a measure of shared pathways and metabolic capacity). Phylogenetic distances were calculated as evolutionary divergence based directly on 16S sequence dissimilarity between all pairs of microbes. We compared this with a “functional” distance calculated as the Jaccard index of non-shared COG families between all pairs of microbial genomes (see Methods). For most pairs of microbes, these measures were highly correlated (Figure 6), not necessarily surprising in that both are influenced by gradual sequence change driven by molecular evolution.

However, several exceptions to this pattern were apparent among the interacting organisms of our study. First, a dramatic separation of phylogenetic and functional distances occurred between positively and negatively associated clades (Figure 6, green lower left vs. red upper right): positive associations were enriched for both phylogenetic and functional similarity, while negative associations showed the inverse pattern. This was partially explained by the basic observation that similar organisms occupy similar niches, as most relationships among similar organisms occurred between clades at different body sites and often between the same clade at two proximal (e.g. oral) or bilateral sites (e.g. left and right retroauricular creases). Conversely, the preference for negative correlations to occur between phylogenetically and functionally different organisms (top right) suggests that the wide range of co-exclusion mechanisms, not only direct competition but also toxin production, environmental modification, and differential niche adaptation [57] required substantial time to develop throughout evolution. Furthermore, interactions in the same body site were primarily negative, suggesting that competition or subniche differentiation were more prevalent in these data than were collaboration or niche sharing.

Exceptions to both of these trends did occur, however, in that related organisms occasionally showed within-site competition, and phylogenetically distant clades sometimes co-occurred. A highlighted example of the former was the negative association between *Bacteroides* and Prevotellaceae family members (also phylum Bacteroidetes) in the gut, reflecting the recurrent tradeoff of this genus with the *Prevotella* as previously linked to enterotypes [15] and/or dietary patterns [58]. As these organisms are closely related, this might reflect alternative metabolic specializations in an otherwise fairly similar gut environment. Conversely, the *Aggregatibacter* were positively associated with members of the highly dissimilar Flavobacteriaceae family in the saliva. As mentioned above, the *Capnocytophaga* (dominant members of the Flavobacteriaceae in these data) are highly metabolically dependent, and positive correlations among organisms are enriched in oral biofilm associated organisms generally (see Figures 3 and 4).

In addition to these on-diagonal outliers (Figure 6), several additional groups of organisms lay off the trend of functional and phylogenetic similarity. That is, some co-occurring/co-exclusive microbes were evolutionarily distant but functionally more similar than expected (below trend), while others were evolutionarily close but functionally distinct (above trend). Several relationships in the upper-left represent single functionally diverse clades that are also widely co-distributed among bilateral or related body sites, such as the Actinobacteria (skin) and Fusobacteriaceae (oral). Such clades' functional distances reflect a relatively high level of within-clade diversity, raising the possibility that a combination of environmental perturbation with a highly structured microenvironment might help to maintain a tension of high functional diversity within a limited phylogenetic range.

In the oral cavity, co-occurrence of such outliers, appearing off the trend of functional and phylogenetic similarity (Figure 6) was limited to low-abundance community members, with some exceptions. Abundant signature taxa such as *Streptococcus* and *Neisseria* often excluded clades with more stringent environmental oxygen requirements regardless of their specific degree of relatedness. *Prevotella* - evolutionarily distant from the *Neisseria* but sharing much functional potential as defined by orthologous gene clusters - exhibited a negative association in the tonsil. Because of their functional similarity, particularly their shared metabolic requirements (both with varying degrees of saccharolytic and proteolytic activities at the species level [59], [60]), this strongly suggests a co-exclusion due to competition for resources, again in addition to their environmental oxygen requirement. At the family level, Pasteurellaceae (composed of *Actinobacillus*, *Aggregatibacter*, *Haemophilus*, and *Pasteurella*) negatively correlate with several other members of the microbiota. Co-occurrence is more common with pairs of functionally similar microbes able to co-exist through combinations of complementarity, commensalism, and cross-feeding of vitamins, amino acids, and other cofactors. Here, the *Prevotella* produce hydrogen, which influences the growth of *Campylobacteria* [61]. *Prevotella* can also be supported by glycine and pyruvate produced from glutathione by *Treponema* species in the periodontal pocket. The most extreme case of organisms both related and correlated were the *Bacteroides* and *Parabacteroides* in the anterior nares. While these clades are not always well-resolved [62], this trend occurred in nine distinct samples (of 282 total), the only ones in which *Bacteroides* occurred nasally at >5% abundance. While a trivial explanation might be misclassification of a small portion of *Bacteroides*, this trend might instead suggest co-occurrence in a metabolic niche that rarely favors either organism but, in the rare occasion of favoring one, permits both.

### Conclusions

We analyzed ecological interactions among bacteria in the human microbiome using 16S marker gene abundance data from the Human Microbiome Project. Our methods for building a microbiome-wide microbial association network combined two complementary approaches: an ensemble of similarity/dissimilarity measures and a compendium of generalized boosted linear models. Relationship significance was assessed using a novel nonparametric approach to compositional data analysis, resulting in a network of co-occurrence and co-exclusion relationships representing potential microbial interactions and incompatibilities within and across body sites. Analysis of the network demonstrated strong organization of the human microbiota into body area niches, mostly among closely related individual body sites representing microbial habitats. A few “hub” microbes were observed to act as signature taxa driving the composition of each microcommunity. Many of these were also the dominant species within a body area, for example *Streptococcus* in the oral cavity and *Bacteroides* in the gut, and these highly abundant taxa also frequently co-occurred as connectors among multiple related body sites. *In vivo* mechanisms were available from prior work for many of these associations, and more generally the phylogenetic and functional relatedness of pairs of co-occurring microbes often explained their associations. In particular, phylogenetically related microbes tended to co-occur at proximal or environmentally similar body sites, while distantly related microbes with shared functional capacities tended to compete.

This microbial association network was described from observational data, and the mechanisms underlying any of these putative interactions may be quite diverse. Positive co-occurrence association types could include nutritional cross-feeding, co-aggregation, co-colonization, signaling pathways, and co-survival in similar environments [4], [63]. Negative exclusion interactions likewise might span toxin or small molecule production, environmental modification (to the detriment of microbial neighbors), immunomodulation, or gross overpopulation of a niche. Ecologically, these data alone do not resolve variations of mutualism, commensalism, amensalism, or predator-prey relationships [4], [63]. Further, all of these ecological relationships, detected here based on microbial abundance patterns across many subjects, are themselves distinct from the biogeographical “co-occurrence” patterns observed by previous studies of individual microbes within subjects [30], [31]. To distinguish between these, future work could include perturbation experiments (e.g. the removal of a species from a defined habitat such as the gut of a gnotobiotic mouse), as these are becoming less difficult to sustain technically [64]. Analytic refinements might instead include defining directionality of relationships in higher-resolution (e.g. temporal) data; for instance, we expect a strict mutualistic relationship (where both partners cannot exist without the other) to be symmetric, whereas the relationship between a prey and a specialized predator is expected to be asymmetric (the prey can occur without its predator, but not vice versa). Negative co-exclusions may have fewer possible initial interpretations, comprising the types of competition outlined above, or they may indicate different, exclusive microbial community states occurring temporally or as linked to host environment [15], [36], [58].

Methodologically, it is again important to emphasize that detecting significant co-occurrences among members of a population assayed as relative abundances can be surprisingly difficult due to compositionality [27]. That is, an absolute increase in one organism's abundance can result in an apparent relative decrease of all other abundances, leading to spurious correlations. Extensive prior work has explained the problem in microbial and macroecological settings [65], and we have mitigated potential issues in these data through our ensemble approach and by principled calculation of significance thresholds using null distributions that incorporate the degree of similarity due solely to compositional effects (see Methods and Text S1). GBLMs were the most distinct method included in this ensemble and share some similarities with recently proposed genetic regulatory network (GRN) reconstruction techniques [66]. GBLMs do provide methodology for discovering GRN-like higher-order interactions in microbial communities, but the accuracy needed to overcome the associated multiple hypothesis testing problems is not yet achievable from available 16S data [67], [68]. We anticipate that future studies with species- or strain-level classification of deep shotgun metagenomic sequences may provide sufficient resolution for more detailed networks including such cooperative microbial associations.

While it might be hoped that easily sampled microbiomes such as the saliva would serve as proxies for e.g. the broader oral microbiome, these results suggest that this is not generally the case. There are few strong correspondences among organisms even at closely related body sites, let alone distal sites, and very few cases where microbial abundance is quantitatively predictable from a proxy sample. In the HMP, this may be a feature of a healthy population, and additional relationships (or disruption of existing ones) might emerge in the presence of disease. Environmental factors that strongly impact the healthy microbiome may additionally not be captured for this population (e.g. diet) and can be further investigated by targeted methodology in future cohorts.

This catalog of microbial co-occurrence and co-exclusion relationships thus provides an initial glimpse of potential mechanisms of community organization throughout the human microbiome. While this computational methodology can be applied to any communities assayed using marker gene sequencing, it is interesting to conclude by noting that the resolution of the resulting network is limited by the specificity of 16S sequence binning. The network discussed here, for example, leverages two specific hypervariable regions for taxonomic classification, each with strengths and weaknesses, and neither individually adequate for sequence classification at the species level [69], [70]. Since it is likely that additional microbial associations will occur at the species or strain level, we anticipate that further community structure will emerge during analysis of metagenomic shotgun sequences taxonomically binned at a finer level of detail. Community shotgun sequencing will also provide functional information regarding metabolism, signaling, and, again, potential physical mechanisms of interaction, which can in turn be matched against complete reference genomes for co-occurring strains. Perturbation analyses in co-culture or, eventually, longitudinal studies in human cohorts will provide an intriguing means of investigating the impact of these microbial “wiring” diagrams on human health.

## Methods

Two complementary approaches, an ensemble of multiple similarity/dissimilarity measures and a compendium of generalized boosted linear models (GBLMs), were used to interrogate significant associations between microbial abundances. These were drawn from 18 body sites assayed by the Human Microbiome Project at two clinical centers using 16S rRNA gene sequencing. Simes method and Benjamini-Hochberg-Yekutieli false discovery rate (FDR) correction were used to combine the resulting networks. From this merged, global network, we summarized overall network properties (degree distribution, modularity, etc.), assessed patterns of microbial connectivity within and among body sites, and identified highly connected (hub) microbes. Phylogenetic and functional distances were calculated based on 16S rRNA gene sequence similarity and shared orthologous gene families, respectively, and combined with the network.

Please see Text S1 for an extensive discussion of the methodology used to assess relationship significance in compositional data, which is presented in summary below.

### 16S data acquisition and processing

The 16S rRNA gene-based dataset of the normal (healthy) human microbiome was made available through the Human Microbiome Project (HMP) and is detailed in [29]. Briefly, it consists of 454 FLX Titanium sequences spanning the V1 to V3 and V3 to V5 variable regions obtained for 239 healthy subjects enrolled at clinical sites in Houston, TX and St. Louis, MO. These cover 18 body sites covering five areas: the oral cavity (nine sites: saliva, tongue dorsum, palatine tonsils, keratinized gingiva, hard palate, buccal mucosa, throat, and sub- and supragingival plaques), the gut (one site: stool), the vagina (three sites: introitus, mid-vagina, and posterior fornix), the nasal cavity (one sample: anterior nares), and the skin (four sites: left and right antecubital fossae and retroauricular creases). Sequences of both 16S windows were processed separately using mothur [71] into phylotypes using the RDP taxonomy as described in [29] and [68], with full protocols also available on the HMP DACC website (http://hmpdacc.org/HMMCP). Genus level and above phylotypes were used for this analysis, for which the datasets from both windows were combined.

This resulted in more than 5,000 samples comprising 910 taxa made available as part of the HMP (http://hmpdacc.org/HMMCP). These were further processed for this study by excluding any phylotype not supported by at least two sequences in at least two samples. Samples were removed as suspect if the most abundant taxon was detected by fewer than 1% of the sequences supporting it in the sample in which it was most abundant, and counts for the remaining 726 taxa were converted to relative abundances in each of the resulting 5,026 samples. Due to potential differences between clinical centers, the dataset was conservatively split into two subsets for further analysis, subjects recruited in Houston (3,296 samples) and those recruited in St. Louis (1,730 samples).

### Generalized Boosted Linear Models

#### GBLM definition and construction

For each resulting dataset, a compendium of generalized boosted linear models (GBLMs) was constructed by selecting all 324 combinations of source body sites *ss* and target sites *ts*. Each GBLM was fit using the abundances of all source taxa st within the source site to predict the abundance of each target taxon *tt* within the target site using a sparse linear model of the form:
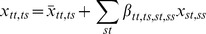



All additional non-leaf clades in the RDP [72] taxonomy (i.e. families, orders, etc. up to the bacterial and archaeal domains) were included as source and target taxa. For *ss* equal to *ts*, i.e. predicting the abundance of a taxon *tt* when the abundances of all taxa in the same body site are known, the abundances of all parent and descendant clades of *tt* were excluded from the available source taxa *st*. That is, when *ss* = *ts* and *tt* was of the form *domain*|*phylum*|*class*|…|*clade*, all source taxa of the form *domain*, *domain*|*phylum*, *domain*|*phylum*|*class*, etc. or of the form *domain*|*phylum*|*class*|…|*clade*|*subclade*, *domain*|*phylum*|*class*|…|*clade*|*subclade*|*subsubclade*, etc. were excluded from the source taxa *st*. This prevented the abundances of *x_tt_*_,*ts*_ from being predicted using abundances *x_st_*_,*ss*_ on which they were directly dependent, while allowing the detection of predictive relationships between distinct clades within the same body site.

The linear model was generalized to include binary categorical target taxa (in this case only gender and ethnicity) using standard logistic regression:
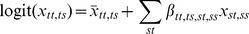



As this is clearly an extremely high-dimensional problem, multiple a priori and post hoc steps were taken to enforce model sparsity and to avoid overfitting for each (*ss*, *ts*, *tt*) tuple. The first of these was to exclude from the available *st* any taxon not correlating with *tt* at a Spearman correlation of nominally p<0.05. The second was to boost linear model fitting rather than attempt to fit all *β_tt_*_,*ts*,*st*,*ss*_ simultaneously [73]. Boosted linear models retain the usual L2 least squares penalty, but are constructed in a manner similar to sparse forward variable selection or the LASSO [74]. *β*s are considered for inclusion in the model one at a time and the parameter minimizing sum of squared error selected and included. However, each subsequent round of parameter fitting operates on the residuals of all previous rounds, thus “upweighting” poorly fit examples, and the inclusion of further non-zero *β*s stops after a fixed number of iterations.

This tuning parameter and the model fitting process was 10-fold cross-validated and selected from the most accurate (by root mean square error for continuous *tt* and AUC for binary) of 50, 100, or 150 boosting iterations using the caret [75] and mboost [76] R packages. This resulted in a compendium of 7±4.9 non-zero parameters *β*, each evaluated with a 10× cross-validated R^2^/AUC and a nominal R^2^/AUC on the full dataset. Final model quality scores were assigned by A) subtracting AUCs below 0.5 from one, since caret does not calculate AUCs directionally, and B) retaining the minimum of the cross-validated and nominal R^2^/AUC. Any continuous model not achieving an R^2^ above zero after adjustment for the number of non-zero parameters (
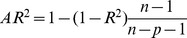
), parameters *p*, training samples *n*) was discarded (55,424 retained).

#### GBLM filtering and significance

Even from cross-validated goodness-of-fit scores, the compositional structure of relative abundance data prevents straightforward assessment of model significance (see Text S1). We thus additionally fit twenty models per (*ss*, *ts*, *tt*) tuple after bootstrap re-sampling the values of *tt* across samples. The AR^2^/AUC of these bootstrap models provided a confidence interval around the observed AR^2^/AUC. Any model for which the 90% confidence interval failed to include the observed AR^2^/AUC was discarded. To construct the null distribution of associations due to compositionality alone, we fit twenty additional models after permuting the values of *tt* across samples and renormalizing them sample-wise, thus retaining compositional effects but breaking true associations. The GBLM was re-fit and the resulting null distribution of AR^2^/AUC values used to assess significance of the true model. The mean and standard deviation of the bootstrap distribution was z-tested against this null distribution using the jointly pooled standard deviation, providing one p-value per model per clinical center. Any model with FDR adjusted p-value greater than 0.05 was discarded (18,286 retained).

### Ensemble scoring

#### Data preprocessing

The 16S data described above were first normalized by dividing each sample by its total phylotype sum. Mislabeled samples were removed [77] and samples were again processed as two subsets, one per clinical center (Houston and St. Louis). These were encoded as a matrix in which each row represented a phylotype in a specific body site and each column represented an individual during one sampling visit. Rows with more than 2/3 zero counts were removed, leaving matrices of 1,217 (Houston) and 1,408 (St. Louis) phylotype-bodysite composite features collected for 248 and 144 subject-visit points, respectively.

#### Ensemble score calculation

We built on composite co-occurrence scores as described in, for example, [78] (for protein functions) and [19] (graph clustering) to find groups of microbial lineages co-existing across a large number of environments. Specifically, we combined four diverse measures in order to overcome two major challenges in the inference of co-occurrence networks, particularly appropriateness of scoring measures to sparse count data and determination of statistical significance. The first is exemplified by the double-zero problem, in which a zero indicates either that an organism is absent or that it is below detection limit [25]; Pearson and Spearman correlations are sensitive to this, while the Bray Curtis dissimilarity is not. The latter issue arises from the need to normalize across samples with unknown absolute abundances, either by relativizing or by downsampling; either procedure results in constrained sample sums, which introduce artificial correlations [27].

We thus employed an ensemble approach combining four diverse measures: two measures of correlation (Pearson and Spearman) and two measures of dissimilarity (Bray-Curtis (BC) and Kullback-Leibler (KLD)). For BC and KLD calculations, rows were divided by their sum prior to computation. Additional measures were considered for our ensemble, including the Hellinger, Euclidean and variance of log-ratios, but these proved to be well-represented by the smaller final ensemble (see Figure S6).

#### Ensemble network building

After running each of the above measures on the two 16S data matrices, one per clinical center, we set measure-specific thresholds as a pre-filter such that each measure contributed 1,000 top-ranking and 1,000 bottom-ranking edges to the network. Edge scores were computed only between clade pairs without parent-descendant relationship (e.g. without pairs of the type Actinobacteridae|Actinomycetales or Actinomycetales|Propionibacterineae) for clades in the same body site. To assign statistical significance to the resulting differently-scaled scores, we first computed edge- and measure-specific permutation and bootstrap score distributions with 1,000 iterations each. In order to address the compositionality issues discussed above [27], we re-normalized the data in each permutation, providing a null distribution that captures the similarity introduced by compositionality alone (see Text S1). We then computed the p-value as above by z-scoring the permuted null and bootstrap confidence interval using pooled variance. P-values were tail-adjusted so that low p-values correspond to co-presence and high p-values to exclusion. For BC and KLD, we did not compute re-normalized permutations, because these measures are intrinsically robust to compositionality [28]. Instead, we calculated their p-values using the bootstrap interval compared to a point null value that was computed by permutation.

Finally, to remove unstable edges, we removed all edges whose score was not within the 95% confidence interval (limited by the 2.5 and 97.5 percentiles) of the bootstrap distribution. Additionally, a number of BC-supported negative links were removed because they were due to abundance profiles including one extreme outlier. This affected the following clades for St. Louis: Actinomycetales in stool, *Corynebacterium* and Corynebacteriaceae in the tonsils, *Lactobacillus* and Lactobacillaceae in the anterior nares and for Houston: an unclassified *Neisseria* in the left retroauricular crease.

### Mitigating the compositional effect in relative abundance analysis

Data summarized as relative abundances are referred to as compositional [28]. Because they sum to one, their elements are not independent and may exhibit spurious correlations regardless of the true underlying relationship. To mitigate this effect, we assessed the significance of our ensemble scores and GBLMs as described above, each by comparing a bootstrap confidence interval around the observed score with a permuted null distribution that includes repeated renormalization to account for compositional effects alone. In each permutation, only the target taxon row was randomized and all samples subsequently renormalized to a constant sum. Because permutation breaks correlation structure while renormalization reintroduces compositionality, a null distribution coupling these elements induces correlation from compositional structure alone. Comparing this null distribution to a standard bootstrap confidence interval around the observed value provided a straightforward nonparametric test of association accounting for compositionality. Simulation studies showed that the bootstrap-renormalization scheme was successful in discounting compositional effects while preserving true correlations (see Text S1).

### Network merging by Simes method and FDR correction

Simes method was used to combine all ten networks (5 methods×2 study centers) into one final network, as it is robust against non-independent tests [79]. A strict intersection of the two clinical centers' networks, rather than a p-value combination, was also examined and found to be over-stringent due to systematic differences in the data (Figure S8). After merging, p-values on each final edge were corrected to FDR q-values using the Benjamini-Hochberg-Yekutieli method and a q-value cutoff of 0.05 was applied. The positivity or negativity of each relationship was determined by consensus voting over all integrated data sources, ranging from −10 (most negative) to 10 (most positive, see Figure 2). Edges with indeterminate directionality (direction score of zero) were removed. Finally, only edges with at least two (out of ten) supporting pieces of evidence were retained.

### Computation of network modularity

The formula by Clauset et al. [32] compares the fraction of edges within input clusters with the fraction of within-cluster edges that would be expected for a randomized network. We clustered the network using the Markov cluster algorithm (MCL) [80] and computed network modularities for a range of inflation parameters. The strongest modularity (0.28) was measured for an inflation of 1.3 and is slightly below the cut-off recommended by Clauset et al.. This modularity was, however, higher than any measured for 100 randomized networks (which preserved node and edge number, but in which edges were randomly re-assigned) and was therefore retained as significant.

### Assessment of significant connectivity density within and among body sites and classes

To assess whether specific body sites were more connected than expected by chance, we repeatedly (1,000 times) selected as many nodes as a body site contains from the global network at random and counted their edge number. This resulted in a distribution of edge numbers for random node sets. To retain only plausibly significant edges for further calculation, we corrected for multiple testing by multiplying the nominal p-value from this distribution with the number of tests carried out and retaining values below 0.05. We repeated this test separately for positive, negative, intra- and cross-edges. For visualization, the network itself was also separated into within-site, within-area, and between-area subsets for further inspection (Figure S9).

### Assessment of relationships between body-site-specific and class-specific clade groups

To assess the interaction strength between body site and clade groups (Figures 4 and 5), the number of relationships between nodes in each group pair was counted and normalized by the group member product, which represents the number of links two groups can potentially form. This was repeated in 1,000 randomized networks (generated as described for the computation of network modularity). A p-value was computed from the count distribution and node group relationships with p-values above 0.05 were discarded, retaining only the fractions of total possible relationships which were significantly higher than those expected by chance.

### Phylogenetic and functional similarity scores

#### Phylogenetic distances

Genome sequences of 1,107 organisms were retrieved from NCBI (http://www.ncbi.nlm.nih.gov/genomes/lproks.cgi, December 2010) and 16S sequences were extracted. These 16S sequences were aligned using MUSCLE 3.8.31 [81] and a full phylogenetic tree reconstructed by maximum likelihood using FastTree 2.1 [82]. A matrix of all pair-wise distances was created from this cladogram and distances between any two nodes (e.g. families, orders, etc.) calculated by taking the median of all distances (as provided in units from FastTree) between all pairs of leaf taxa descending from the two nodes.

#### Functional distances

Functional complements of the same genomes were summarized using COG [83] families as assigned by NCBI annotations. This resulted in an abundance matrix with 4,685 columns (corresponding to COG families) and one row per genome. Columns summing to less than 10% of the number of genomes were removed, resulting in 3,514 usable COG families. Pairwise scores between genomes were calculated using Jaccard index, with distances for higher-level clades computed using medians in the same manner as described above. Final functional distances were represented as the Jaccard index of non-shared COG families between pairs of genomes.

## Supporting Information

Figure S1 **Significant co-occurrence and co-exclusion relationships among the abundances of clades in the human microbiome.** The network displays all significant phylotype associations within and across the 18 body sites sampled by the HMP. Nodes represent phylotypes (colored according to the body site in which they occur) whereas edges represent significant relationships between phylotypes. Edge thickness reflects the strength of the relationship, and edge color its directionality (green co-occurrence, red co-exclusion).
(PDF)

Figure S2 **Markov clustering of the complete phylotype network.** Markov-clustered network (inflation parameter: 1.3). When clustering the cross-body site network with this inflation parameter giving optimal modularity, the network splits into the set of depicted clusters (75 in total). Many of them are specific to body sites (stool, anterior nares) or areas (mouth, vagina, skin).
(TIF)

Figure S3 **Cluster coefficients of association networks within individual body sites and clades.** Average cluster coefficients (computed with tYNA [85]) of body-site-specific (A) and class-specific (B) sub-networks. The “cliquishness” of each node within a body site or class is expressed by the average cluster coefficient, which is higher when the neighbors of each node are also connected among themselves. It can be zero if none of the nodes in the sub-network has inter-linked neighbors. The cluster coefficient was computed for all edges of a sub-network (gray bars) and for positive (green bars) and negative edges (red bars) separately. Strikingly, almost none of the negative-edge-only sub-networks had cluster coefficients above zero. In the case of the negative class sub-networks, this is a consequence of the low number of intra-class negative edges (see Figure 3E). If a negative-edge-only sub-network has a cluster coefficient of zero, it means that neighbors of a node are either not interconnected at all or that they are interconnected only by positive edges. Within the body sites, groups of phylotypes linked by negative edges likely reflect alternative communities. Members of these communities are linked among themselves by positive edges. Thus, if the positive edges are removed, the neighbors of negative nodes are no longer interlinked and the average cluster coefficient becomes zero. The high positive-edge-only cluster coefficients in classes correspond well to the high positive intra-edge number in these classes (see Figure 3E) and mean that if one member of the class is present in an individual, the other members are also likely present.
(TIF)

Figure S4 **Co-exclusion of** ***Tannerella*** **and** ***Streptococcus*** **in the subgingival plaque.** The anaerobic and proteolytic *Tannerella* requires a lower pO_2_ than *Streptococcus*, while *Streptococcus* is an asaccharolytic colonizer of the tooth surface that uses sugars as its primary source of carbon [49], [50]. Between the supragingival and the subragingival plaques, as well as within the subgingival plaques, a gradient of nutrition and oxygen is present. The gradual drop of the abundance of *Tannerella* as the streptococci increase reflects the continuous nutritional and oxygen gradient between and within the supragingival and the subgingival biofilms.
(TIF)

Figure S5 **Abundances of 18 putative associations between oral and gut microbes.** Quality control plots of the raw data for all putatively significant oral/gut microbial associations showed no strong evidence for microbial transfer from the oral cavity along the digestive tract at the available level of detection. For GBLM associations, plots show predictions from the full linear model (x axis) against observed values (y axis) with the line of unity drawn as a guide, with data from the two clinical centers distinguishable by color (orange = Baylor College, purple = Washington University). None of the significant associations proved to be substantially robust from any of the nine oral body sites to gut microbes.
(TIF)

Figure S6 **Repeatability of network inference using seven individual similarity/dissimilarity measures with the Houston data subset.** The 2,000 most extreme (1,000 top- and bottom-scoring) edges were computed for each measure in the Houston sample subset. Measure similarity was then computed as the Jaccard index of edge overlap. Abbreviations: KLD = Kullback-Leibler dissimilarity, Var-Log = variance of log-ratios, a measure recommended by Aitchison to compute associations between parts of compositions [28].
(TIF)

Figure S7 **Agreement between association networks produced by individual similarity measures and datasets.** Heat map depicting the edge overlap as measured by the Jaccard index between the different methods and sample sets (Houston versus St. Louis) employed. By design from our ensemble of scoring measures, which were chosen to capture different types of microbial co-occurrences, the networks are first grouped by measure into correlations (Pearson, Spearman), GBLMs, and dissimilarities (KLD, Bray-Curtis). Each of these clusters then differentiated further according to sample set (e.g. Spearman and Pearson in Houston versus Spearman and Pearson in St. Louis).
(TIF)

Figure S8 **Intersection of networks generated independently for the Houston and St. Louis clinical center sample subsets.** Our co-occurrence/exclusion network built on the combination of p-values for microbial interaction from 10 distinct networks, generated by five methods in each of two sample subsets from the HMP's Houston and St. Louis clinical centers. We examined the feasibility of treating these two clinical centers as replicates rather than semi-independent observations by performing a hard intersection, i.e. applying Simes method to each set of five methods separately and retaining only the edges significant in both. This intersection retained only 499 nodes and 938 edges, almost all of which (902, 96%) were contained in the complete network. This represents approximately 30% of the edges in the complete network, with the remainder made up of significant relationships confidently detected at only one clinical center. As the two clinical centers differed systematically in minor technical details such as input DNA concentration and chimerism during 16S sequencing [68], treating these as non-independent but non-replicate observations likely represents a more complete model of the HMP data's microbial co-occurrence and exclusion networks.
(TIF)

Figure S9 **Co-occurrence and exclusion relationships within each body site, within body areas, and between body areas.** Sub-networks consisting of (A) 1,409 edges among clades within one body site, (B) 1,552 edges spanning body sites within the same area (such as the oral cavity or vagina), and (C) 44 interactions between distinct body areas.
(TIF)

Table S1 **Complete network of co-occurrence and co-exclusion relationships among clades in the human microbiome.** Each row represents an association between two clades in specific body sites, together with their supporting methods, sign of association (positive or negative), and FDR-corrected significance.
(XLSX)

Table S2 **Over-representation of associations between organisms in body-site- and clade-specific subnetworks.** Over-representation of edges within and between body-site- and clade-specific sub-networks was assessed at the class level by computing edge numbers in 1,000 randomly selected sub-networks of equal node number to the sub-network(s) of interest. This table gives the Bonferroni-corrected p-value and the median edge number of the random sub-networks in brackets in the form (total, p-value, random). Nominally significant values below 0.05 are highlighted in green. Tongue dorsum is the only body site with a significant over-representation of negative edges, and the Bacilli, Bacteroidia and Fusobacteria are the only clades with significant negative relationships.
(XLSX)

Table S3 **Negative and positive association degrees of individual body site clades' nodes.** For each clade in the network, the number of total, positive and negative links to other clades is listed in descending order. In addition, each clade's number of intra- and cross-body-site links is given. The top hub nodes highlighted in Figure 3A thus appear as the first four table rows.
(XLSX)

Text S1 **Description of ReBoot.** Detailed description of the permutation-renormalization and bootstrap (ReBoot) method, which is designed to assess the significance of associations in compositional data.
(DOCX)
