# Supplementary material for: Microbial Co-occurrence Relationships in the Human Microbiome
Source: PLoS Comput Biol. 2012 Jul 12;8(7):e1002606. doi: 10.1371/journal.pcbi.1002606 (PMC3395616; doi:10.1371/journal.pcbi.1002606)
Supplement: Figure S1 — Significant co-occurrence and co-exclusion relationships among the abundances of clades in the human microbiome. The network displays all significant phylotype associations within and across the 18 body sites sampled by the HMP. Nodes represent phylotypes (colored according to the body site in which they occur) whereas edges represent significant relationships between phylotypes. Edge thickness reflects the strength of the relationship, and edge color its directionality (green co-occurrence, red co-exclusion). (PDF) [file pcbi.1002606.s001.pdf]

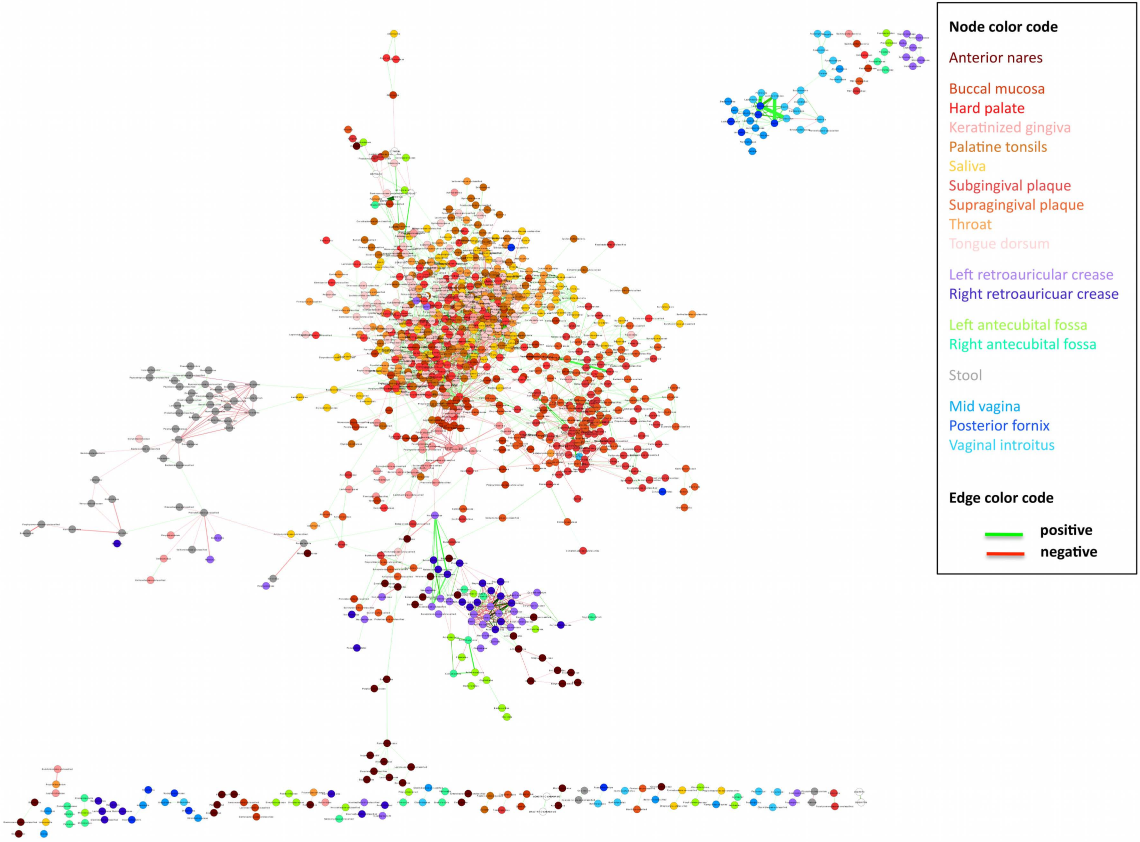
Node color code
Anterior nares
Buccal mucosa
Hard palate
Keratinized gingiva
Palatine tonsils
Saliva
Subgingival plaque
Supragingival plaque
Throat
Tongue dorsum
Left retroauricular crease
Right retroauricuar crease
Left antecubital fossa
Right antecubital fossa
Stool
Mid vagina
Posterior fornix
Vaginal introitus
Edge color code
positive
negative
