# Supplementary figures and images for: Microbial Co-occurrence Relationships in the Human Microbiome
Source: PLoS Comput Biol. 2012 Jul 12;8(7):e1002606. doi: 10.1371/journal.pcbi.1002606 (PMC3395616; doi:10.1371/journal.pcbi.1002606)

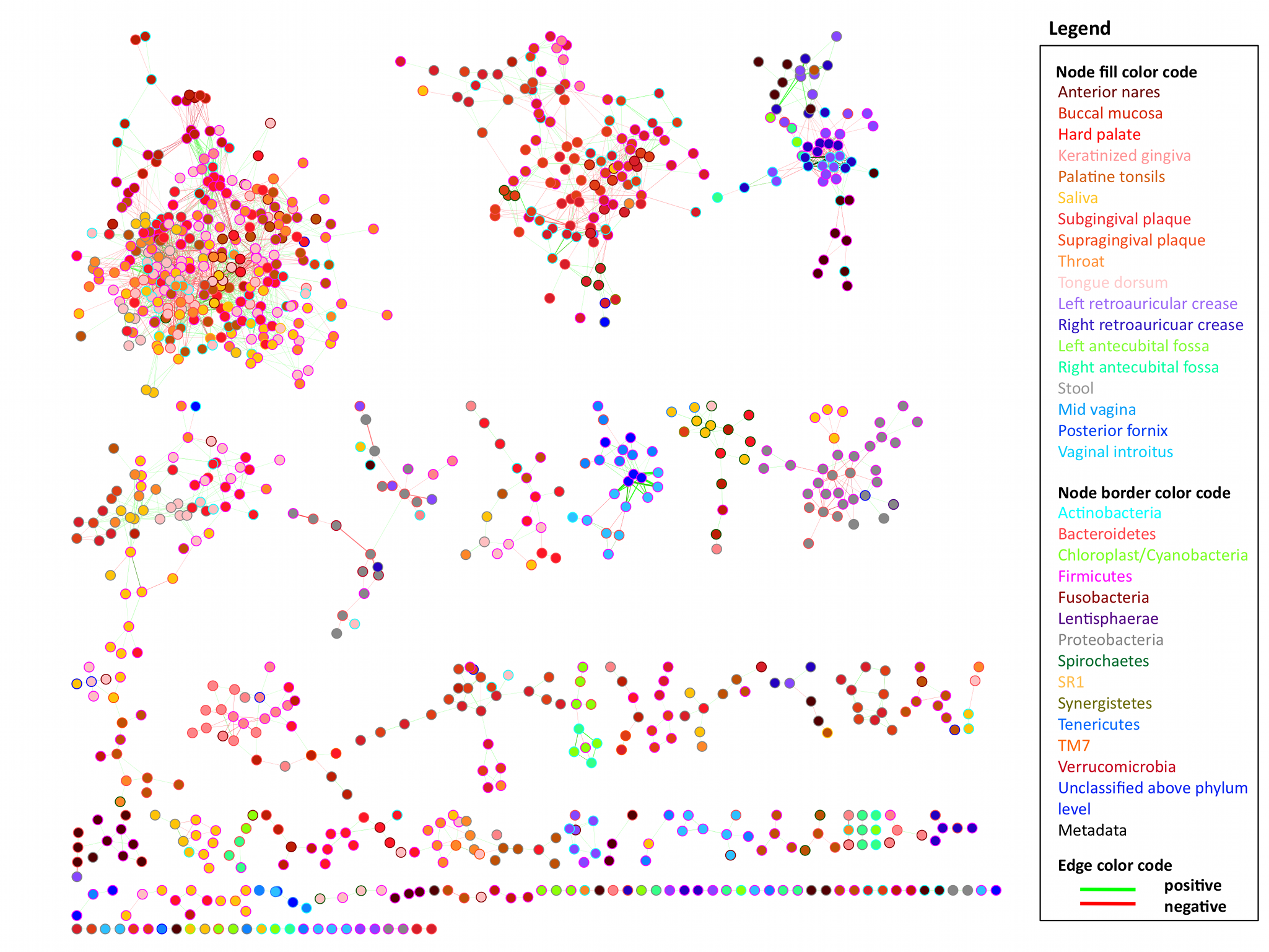

Supplement: Figure S2 — Markov clustering of the complete phylotype network. Markov-clustered network (inflation parameter: 1.3). When clustering the cross-body site network with this inflation parameter giving optimal modularity, the network splits into the set of depicted clusters (75 in total). Many of them are specific to body sites (stool, anterior nares) or areas (mouth, vagina, skin). (TIF) [file pcbi.1002606.s002.tif]

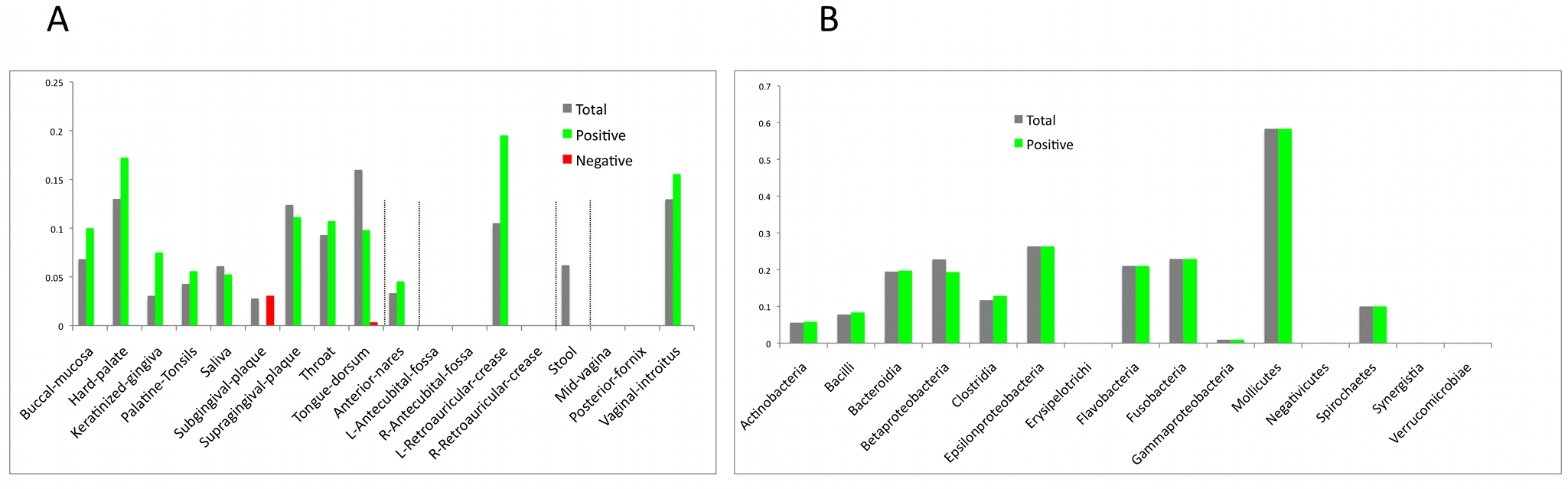

Supplement: Figure S3 — Cluster coefficients of association networks within individual body sites and clades. Average cluster coefficients (computed with tYNA [85]) of body-site-specific (A) and class-specific (B) sub-networks. The “cliquishness” of each node within a body site or class is expressed by the average cluster coefficient, which is higher when the neighbors of each node are also connected among themselves. It can be zero if none of the nodes in the sub-network has inter-linked neighbors. The cluster coefficient was computed for all edges of a sub-network (gray bars) and for positive (green bars) and negative edges (red bars) separately. Strikingly, almost none of the negative-edge-only sub-networks had cluster coefficients above zero. In the case of the negative class sub-networks, this is a consequence of the low number of intra-class negative edges (see Figure 3E). If a negative-edge-only sub-network has a cluster coefficient of zero, it means that neighbors of a node are either not interconnected at all or that they are interconnected only by positive edges. Within the body sites, groups of phylotypes linked by negative edges likely reflect alternative communities. Members of these communities are linked among themselves by positive edges. Thus, if the positive edges are removed, the neighbors of negative nodes are no longer interlinked and the average cluster coefficient becomes zero. The high positive-edge-only cluster coefficients in classes correspond well to the high positive intra-edge number in these classes (see Figure 3E) and mean that if one member of the class is present in an individual, the other members are also likely present. (TIF) [file pcbi.1002606.s003.tif]

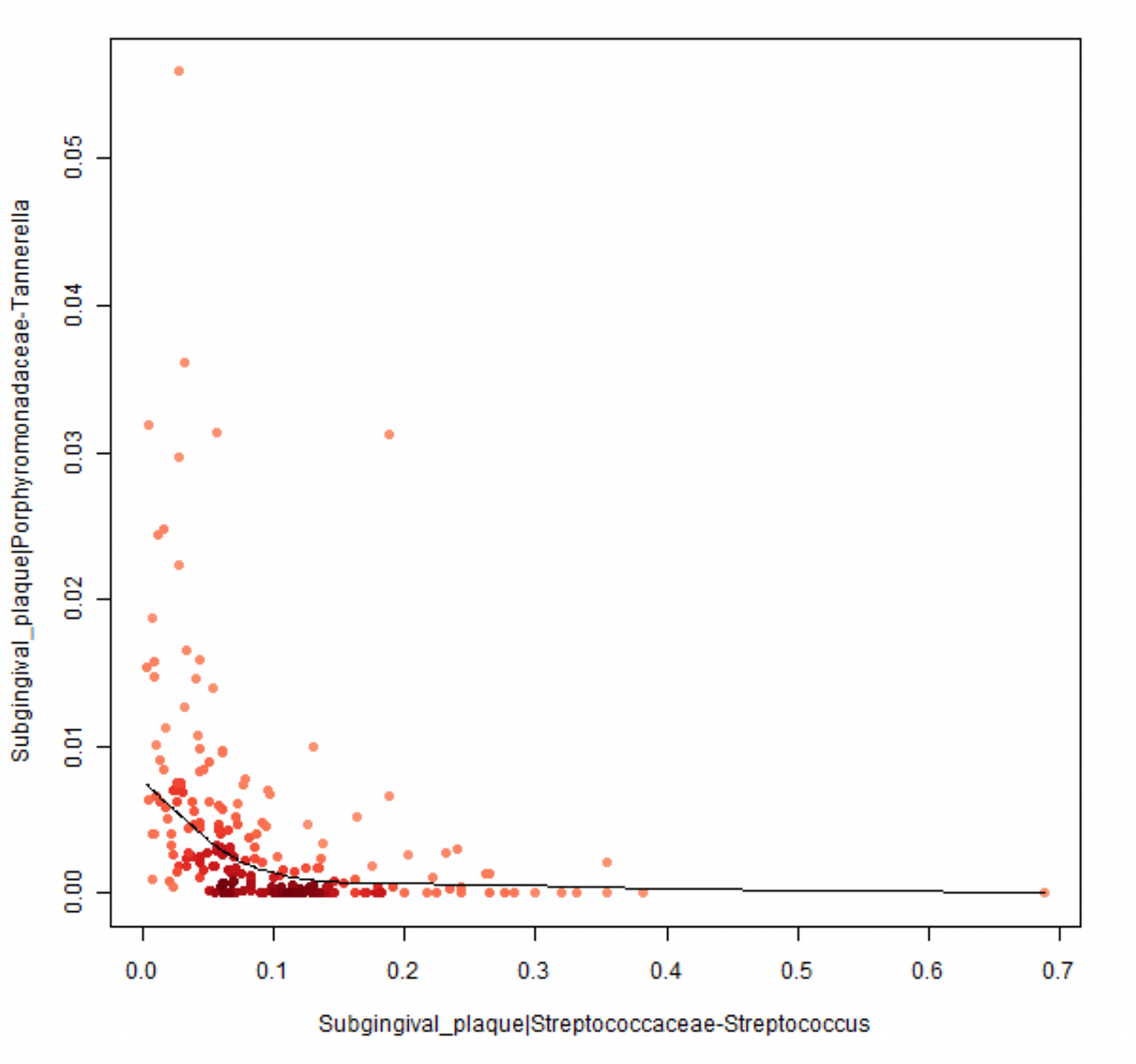

Supplement: Figure S4 — Co-exclusion of Tannerella and Streptococcus in the subgingival plaque. The anaerobic and proteolytic Tannerella requires a lower pO2 than Streptococcus, while Streptococcus is an asaccharolytic colonizer of the tooth surface that uses sugars as its primary source of carbon [49], [50]. Between the supragingival and the subragingival plaques, as well as within the subgingival plaques, a gradient of nutrition and oxygen is present. The gradual drop of the abundance of Tannerella as the streptococci increase reflects the continuous nutritional and oxygen gradient between and within the supragingival and the subgingival biofilms. (TIF) [file pcbi.1002606.s004.tif]

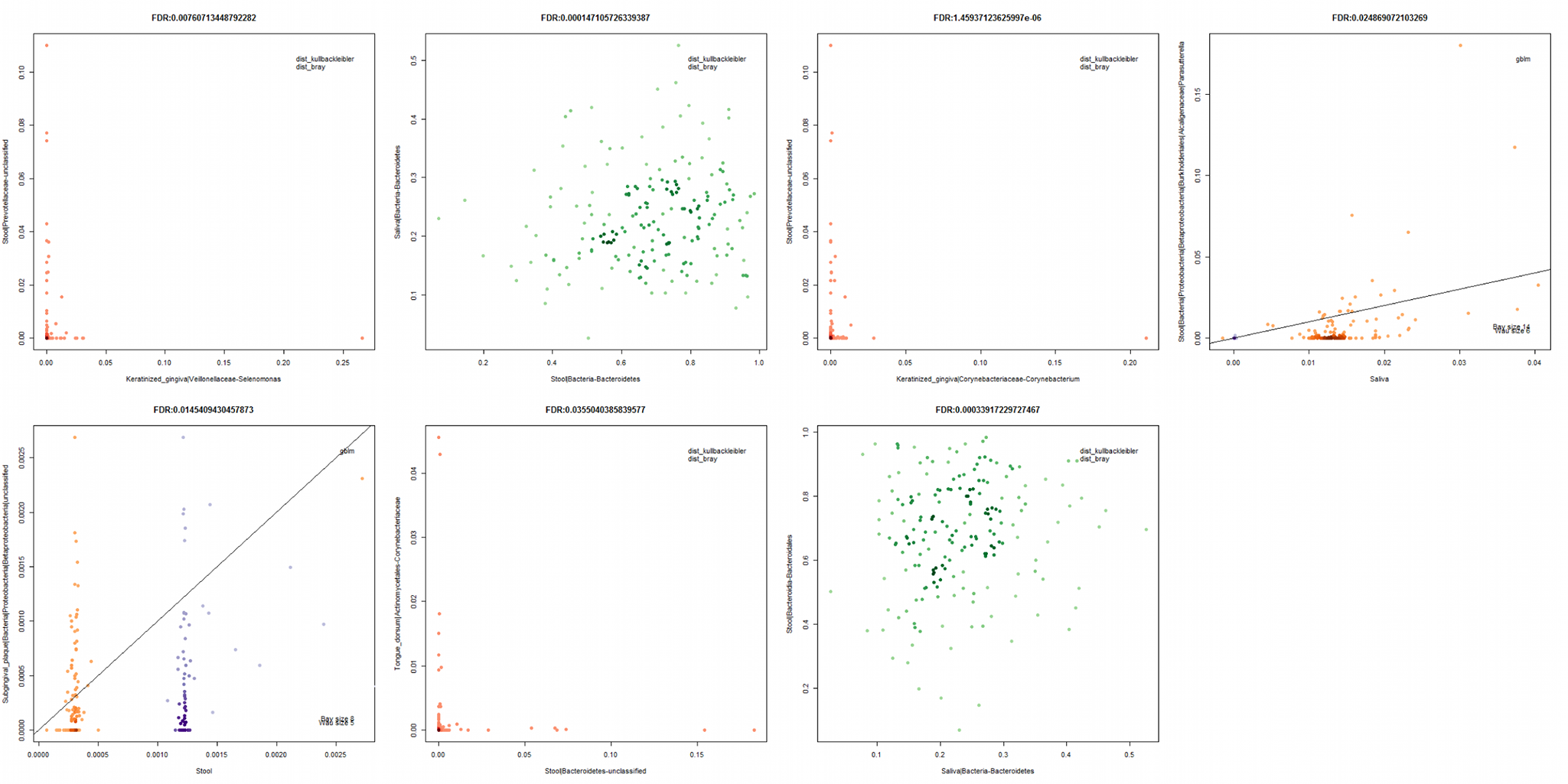

Supplement: Figure S5 — Abundances of 18 putative associations between oral and gut microbes. Quality control plots of the raw data for all putatively significant oral/gut microbial associations showed no strong evidence for microbial transfer from the oral cavity along the digestive tract at the available level of detection. For GBLM associations, plots show predictions from the full linear model (x axis) against observed values (y axis) with the line of unity drawn as a guide, with data from the two clinical centers distinguishable by color (orange = Baylor College, purple = Washington University). None of the significant associations proved to be substantially robust from any of the nine oral body sites to gut microbes. (TIF) [file pcbi.1002606.s005.tif]

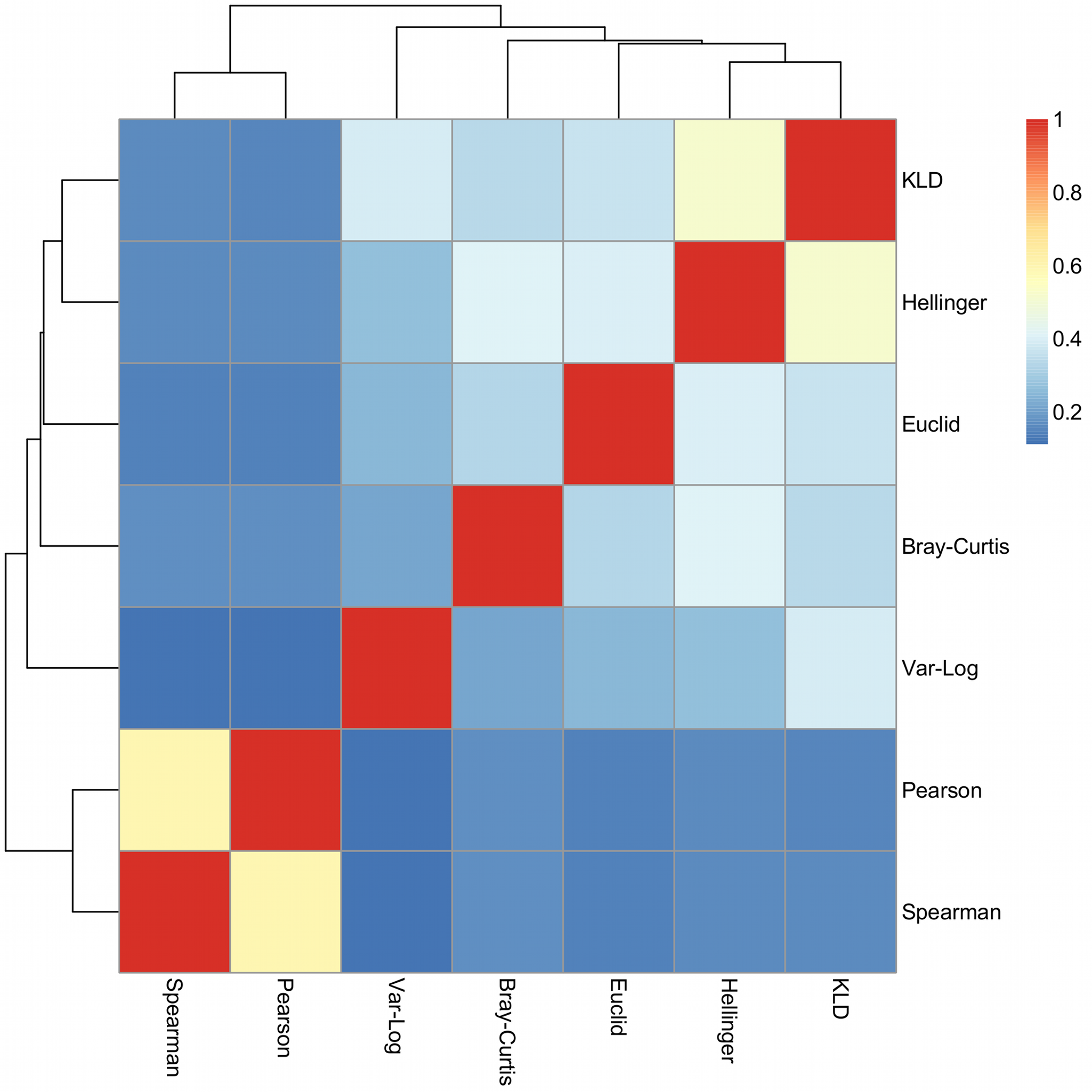

Supplement: Figure S6 — Repeatability of network inference using seven individual similarity/dissimilarity measures with the Houston data subset. The 2,000 most extreme (1,000 top- and bottom-scoring) edges were computed for each measure in the Houston sample subset. Measure similarity was then computed as the Jaccard index of edge overlap. Abbreviations: KLD = Kullback-Leibler dissimilarity, Var-Log = variance of log-ratios, a measure recommended by Aitchison to compute associations between parts of compositions [28]. (TIF) [file pcbi.1002606.s006.tif]

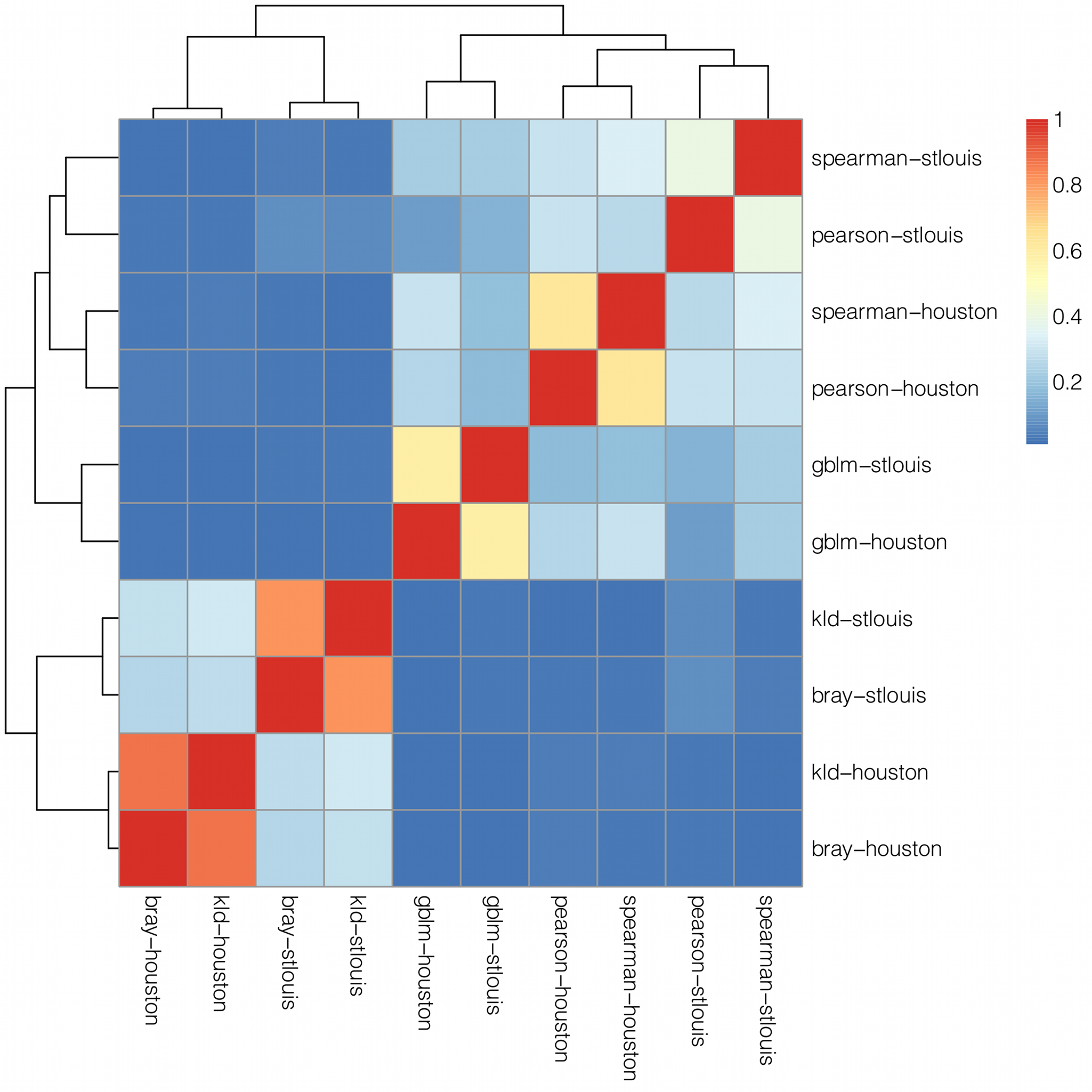

Supplement: Figure S7 — Agreement between association networks produced by individual similarity measures and datasets. Heat map depicting the edge overlap as measured by the Jaccard index between the different methods and sample sets (Houston versus St. Louis) employed. By design from our ensemble of scoring measures, which were chosen to capture different types of microbial co-occurrences, the networks are first grouped by measure into correlations (Pearson, Spearman), GBLMs, and dissimilarities (KLD, Bray-Curtis). Each of these clusters then differentiated further according to sample set (e.g. Spearman and Pearson in Houston versus Spearman and Pearson in St. Louis). (TIF) [file pcbi.1002606.s007.tif]

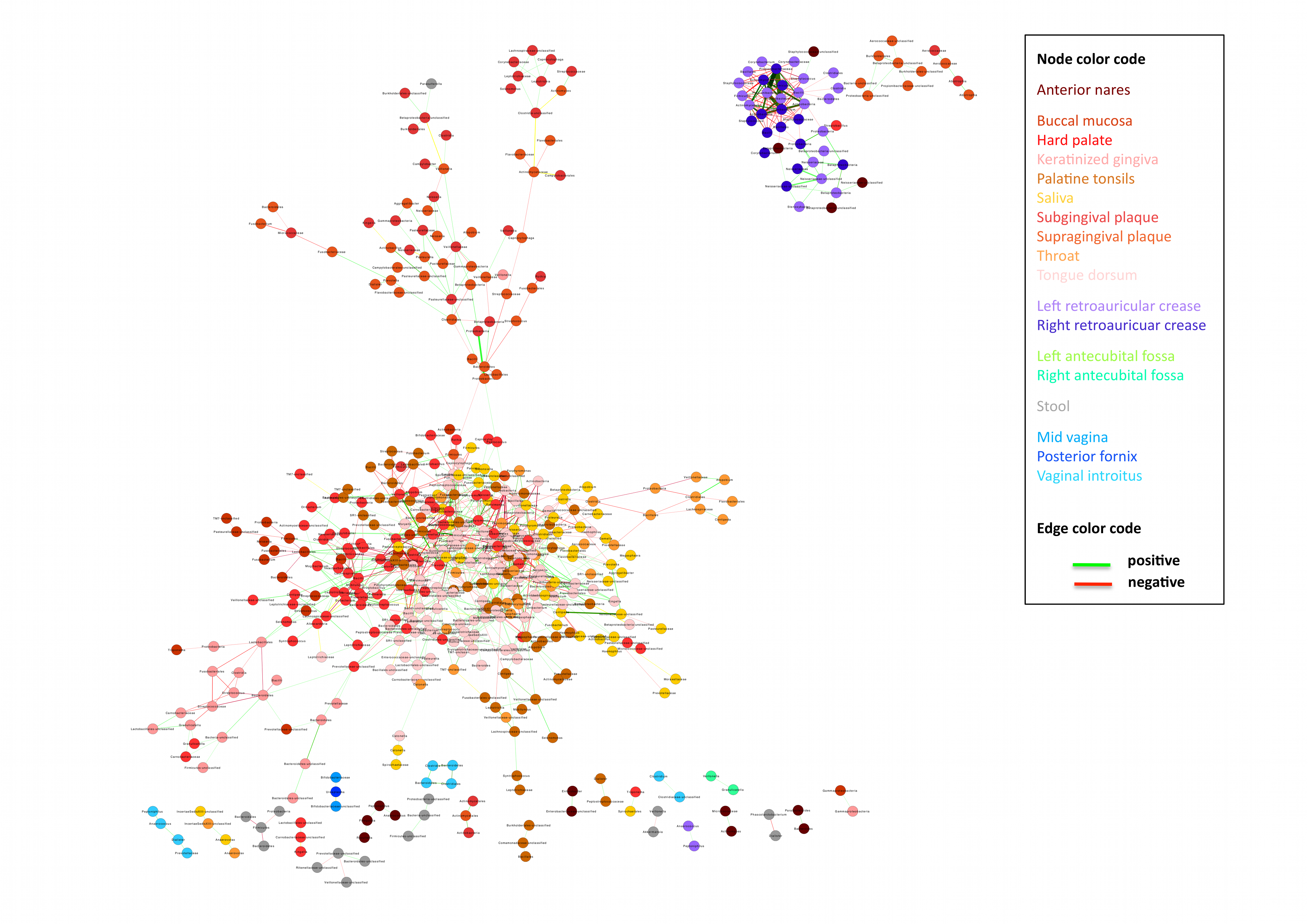

Supplement: Figure S8 — Intersection of networks generated independently for the Houston and St. Louis clinical center sample subsets. Our co-occurrence/exclusion network built on the combination of p-values for microbial interaction from 10 distinct networks, generated by five methods in each of two sample subsets from the HMP's Houston and St. Louis clinical centers. We examined the feasibility of treating these two clinical centers as replicates rather than semi-independent observations by performing a hard intersection, i.e. applying Simes method to each set of five methods separately and retaining only the edges significant in both. This intersection retained only 499 nodes and 938 edges, almost all of which (902, 96%) were contained in the complete network. This represents approximately 30% of the edges in the complete network, with the remainder made up of significant relationships confidently detected at only one clinical center. As the two clinical centers differed systematically in minor technical details such as input DNA concentration and chimerism during 16S sequencing [68], treating these as non-independent but non-replicate observations likely represents a more complete model of the HMP data's microbial co-occurrence and exclusion networks. (TIF) [file pcbi.1002606.s008.tif]

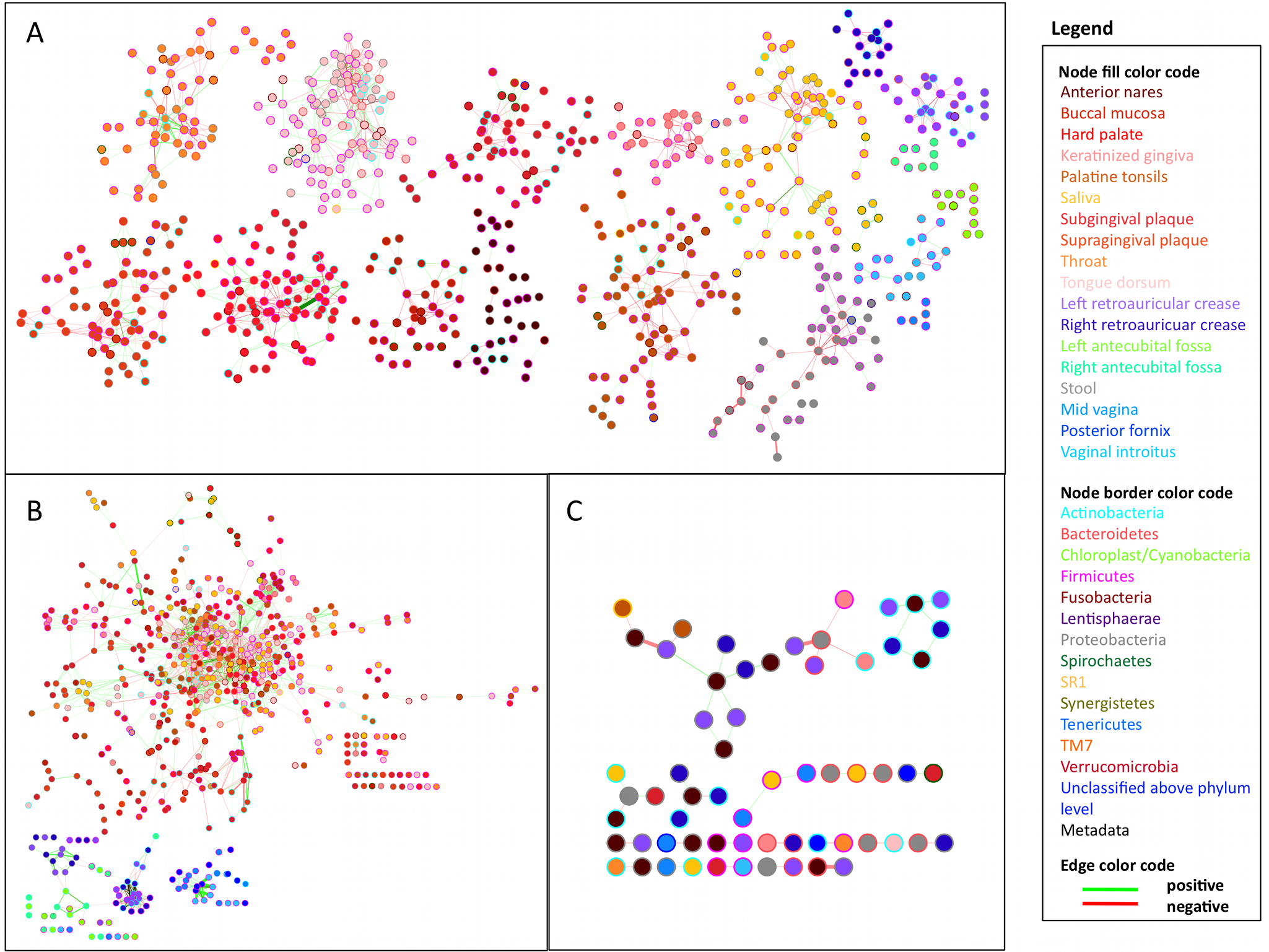

Supplement: Figure S9 — Co-occurrence and exclusion relationships within each body site, within body areas, and between body areas. Sub-networks consisting of (A) 1,409 edges among clades within one body site, (B) 1,552 edges spanning body sites within the same area (such as the oral cavity or vagina), and (C) 44 interactions between distinct body areas. (TIF) [file pcbi.1002606.s009.tif]
