# Supplementary material for: Microbial Co-occurrence Relationships in the Human Microbiome
Source: PLoS Comput Biol. 2012 Jul 12;8(7):e1002606. doi: 10.1371/journal.pcbi.1002606 (PMC3395616; doi:10.1371/journal.pcbi.1002606)
Supplement: Text S1 — Description of ReBoot. Detailed description of the permutation-renormalization and bootstrap (ReBoot) method, which is designed to assess the significance of associations in compositional data. (DOCX) [file pcbi.1002606.s013.docx]

# Supplemental Methods

## Accounting for Compositional Structure in Relative Abundance Data

Data summarized as relative, rather than absolute, abundances such as 16S profiling is known to be challenging to analyze due to compositional measurements. Specifically, since relative abundances are normalized to sum to one (or equivalently rarefied to an arbitrary constant total), an increase in one relative abundance must be accompanied by a compositional decrease in another. This is true even for raw sequence read counts due to the fixed depth of sequencing, which samples proportionally from a total underlying population. Assessing correlation between compositional data by traditional measures such as Pearson’s correlation can lead to spurious correlations [[1](#_ENREF_1)]. Thus, we developed a novel methodology that mitigates the compositional effect in data while assessing significance of an association. In particular, we compare the distribution of correlations from bootstrap sampled data, which represents the confidence interval of the observed correlation, with the null distribution of correlations from renormalized permuted data, which represents the correlation structure arising purely from the compositionality.

In general, assessing significance of an association (e.g. correlation between two vectors) involves comparing an observed value to an appropriate null distribution. Permutation, which breaks any true association in the data, is a common approach for constructing the null distribution. Here, we show that simple permutation does not give an appropriate null distribution for compositional data, as it breaks compositionality and provides anti-conservative estimates. However, appropriate compositional structure can be re-introduced to permuted data by renormalization, and a null distribution can be obtained from the resulting permutation-renormalization scheme. This approach can be further improved by using bootstrapped confidence intervals for the observed value as opposed to the point observation itself, since the bootstrap process scales the variance of the test according to the severity of compositional effect as manifested through the signal-to-noise ratio.

### Compositionality leads to spurious correlation and loss of information

As discussed previously [[1](#_ENREF_1)], relative abundance data which sum to a constant (e.g. one) can exhibit spurious correlation. For example, in Figure SM1, the absolute (A and B) and relative (C) abundances of four microbes (b1-4) are shown. Microbes b1 and b2 are uncorrelated in Figure SM1A, but the relative abundance (Figure SM1C) shows negative correlation between them because of the compositional effect introduced through normalization. It is then important to account for the compositional effect in assessing correlation between abundance data.

Unfortunately, some true correlation may not be possible to recover. In the example shown in Figure SM1, although the relationship between b1 and b2 is different in A and B, the two different absolute abundance datasets produce exactly the same relative abundance profiles, masking the difference in the true correlation structure. Thus, in some cases, it may be impossible to uncover the true underlying correlation given the relative abundance data alone. Hence, it is important to stress that because only the relative abundances were observed in our HMP 16S dataset, some true correlation between microbes may not be possible to elicit. And in evaluating the performance of our method, we need to bear in mind that our aim is to mitigate the effect of compositionality and lessen the confidence in correlations that are likely results of the compositional structure rather than to completely recover all true correlations.


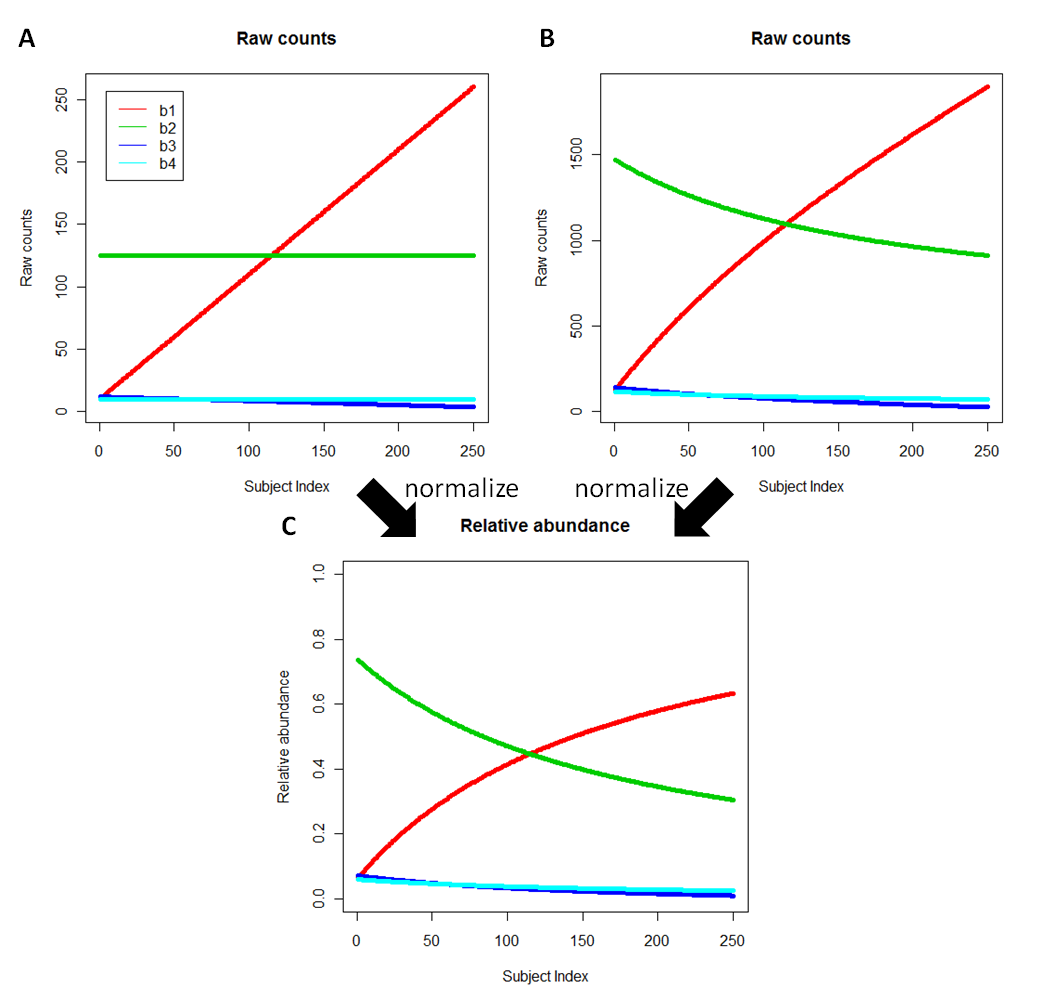


Figure SM1 Compositionality induces spurious correlation and causes loss of information. Consider two possible absolute abundance profiles from collections of microbial communities including taxa b1 through b4. 250 synthetic samples (A) and (B) represent absolute abundances (read counts) that, upon normalization into relative abundance (C), produce identical values from which it is impossivle to recover the original information. Hence it is important to note that in even the best-handled relative abundance data, there may be some correlation that cannot completely be recovered.


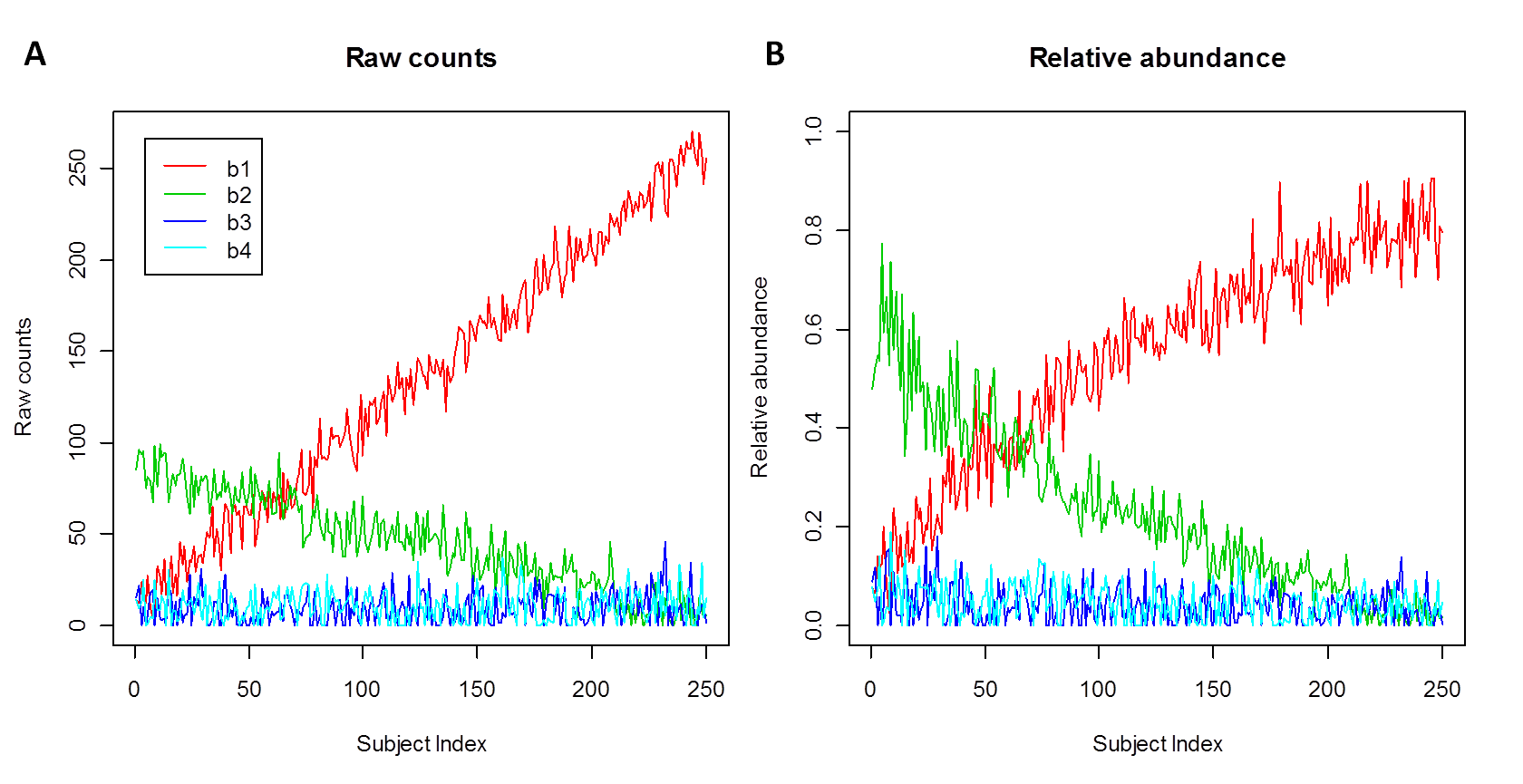


**C**


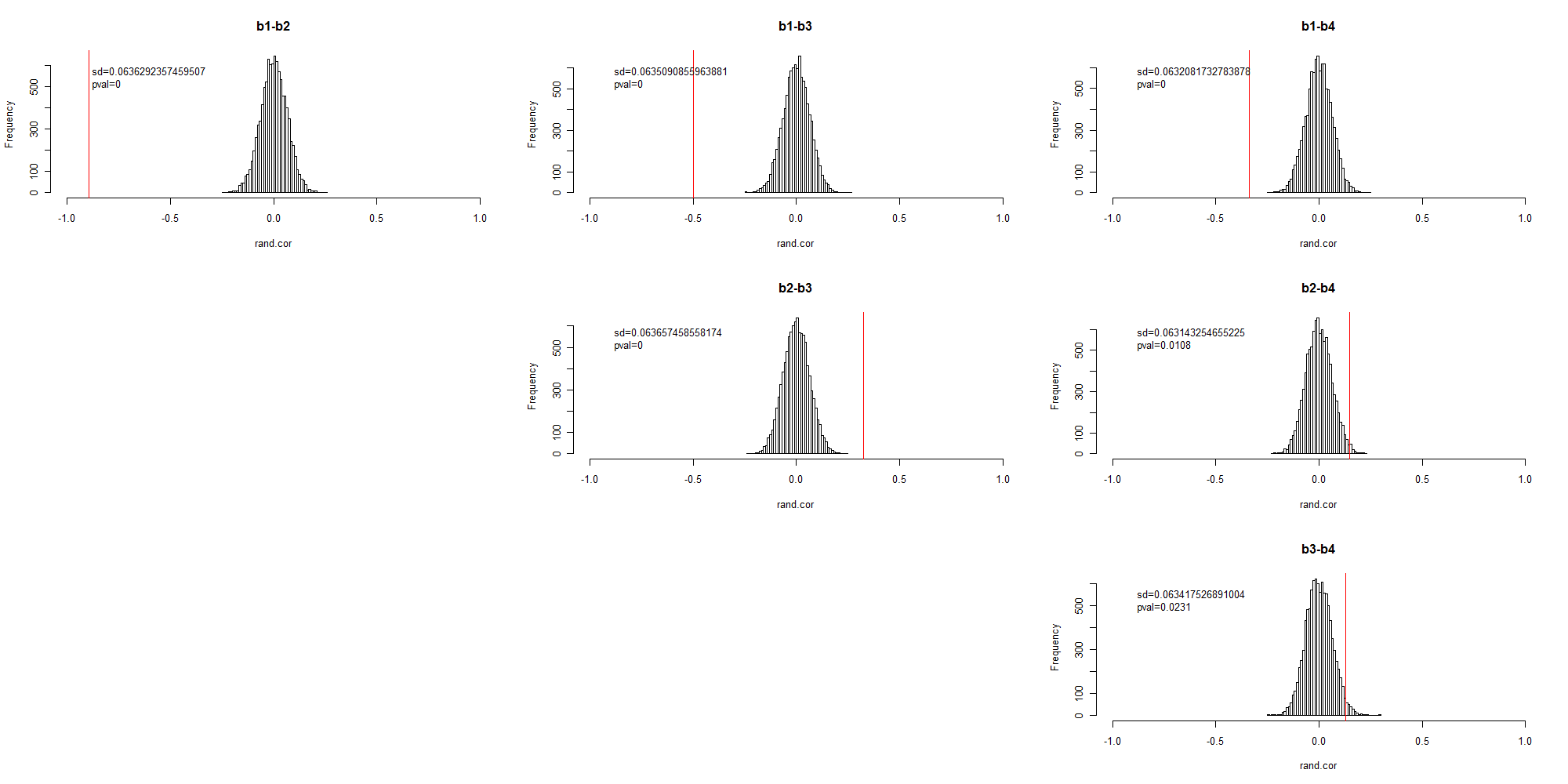


Figure SM2 Permutation testing alone cannot distinguish between true and spurious correlations in compositional data. (A-B) Simulated absolute and relative microbial abundances. (C) Pairwise correlations (vertical red lines) based on the relative abundance in (B) for each pair of synthetic microbes. These are accompanied by permutation-based null distributions, standard deviations, and z-test p-values, all of which are highly (and incorrectly) significant due to compositional effects.

### Standard permutation tests result in false positive correlations

One standard procedure to evaluate the significance of a correlation is a permutation test. Unfortunately, the permutation test cannot distinguish spurious compositional correlations, as the permutation process removes all compositional effects and generates a highly anti-conservative null distribution. As demonstrated in an example in Figure SM2, the permutation test declares all pairwise correlation in a synthetic dataset to be highly significant. This is because the location and scale of the permutation null distribution does not reflect the compositional structure. The distribution always centers at zero with constant standard deviation. A desired null distribution should vary the location to reflect the portion of eventual correlation attributable to compositionality alone and vary the scale to reflect measurement scale based on variation in the absolute abundance.

### The Permutation-Renormalization and Bootstrap (ReBoot) Method

We propose ReBoot: Permutation-Renormalization and Bootstrap Method, a procedure to construct a null distribution that reflects the compositional effect in the abundance data. As shown in Figure SM3, the method starts with the relative abundance data and consists of two steps: constructing a compositionality-aware null distribution and comparing this with a bootstrap confidence interval around the observed correlation. These steps proceed as follows:

#### Constructing the compositional null distribution

For each pair of microbes:

1. Permute the relative abundance of the microbes
2. Renormalize the permuted abundance by summing over each sample and dividing the abundance in that sample by the sample sum
3. Calculate the correlation between the renormalized permuted relative abundances of the two microbes
4. Repeat (1)-(3) *N* times to obtain the compositional null distribution

#### Constructing the bootstrap confidence interval

For each pair of microbes:

1. Sample with replacement the sample indices and construct a bootstrap resampled dataset
2. Calculate the correlation between the bootstrapped relative abundances of the two microbes
3. Repeat (1)-(2) *B* times to obtain the bootstrap distribution, which represents the confidence interval around the observed correlation

Finally, compare the compositional null distribution and the bootstrap distribution by z-test with the variance pooled from both distributions using an equally weighted unbiased least square estimate.


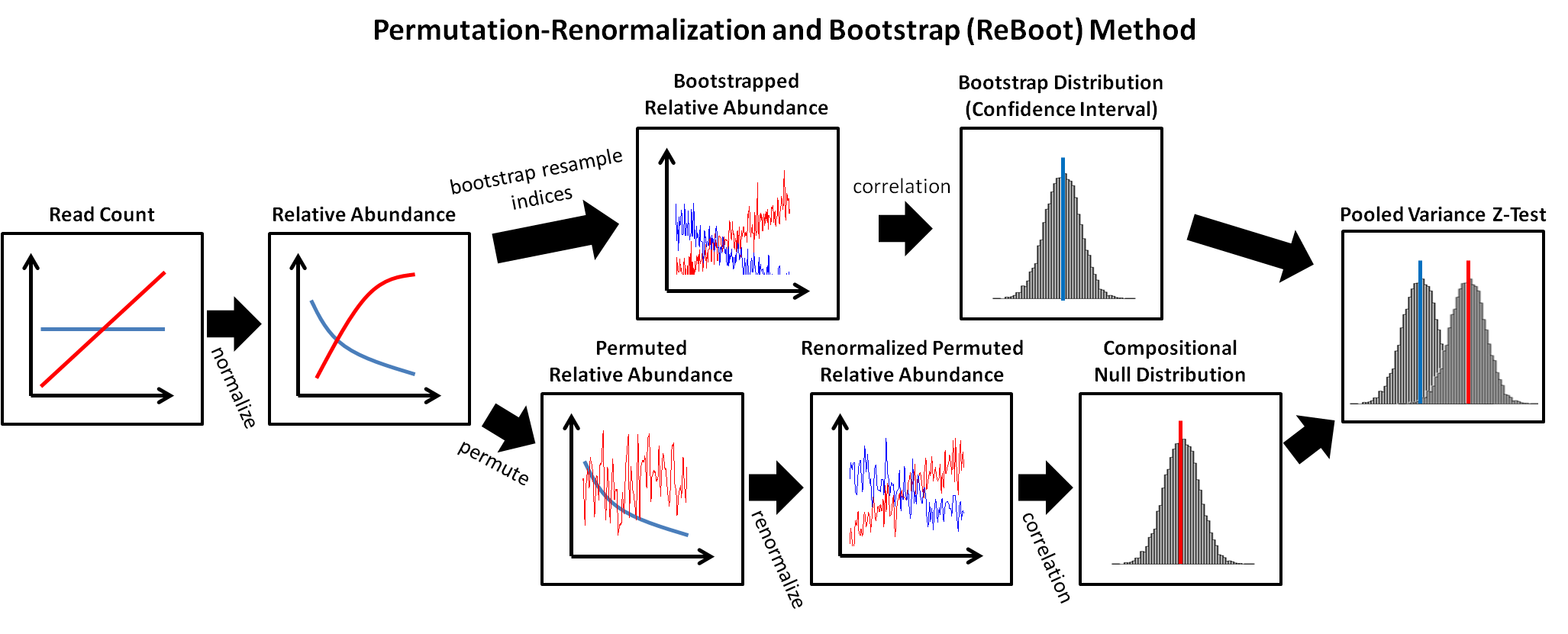


Figure SM3 The Permutation-Renormalization and Bootstrap (ReBoot) Method. From the relative abundance data, we construct (1) bootstrap confidence interval around the observed correlation and (2) the null distribution that represent the correlation due to compositionality alone. Contrasting the two distributions through z-test with pooled variance, an appropriate significance level of the observed correlation can be assessed.

#### Intuition

The bootstrap distribution represents the confidence interval around the observed correlation, which will be wider for less abundant organisms due to lower signal-to-noise ratios. The permutation-renormalization null distribution represents correlation due solely to the compositional effect (see the detailed explanation below). The significance of associations over and above those expected from compositionality alone can thus be evaluated by comparing these two distributions. The null hypothesis to be tested is that the two distributions have the same mean, making z-test an appropriate comparison. The variance pooled with equal weight from both distributions provides an unbiased least squares estimate.

### ReBoot method works well on simulated data

We demonstrated the performance of the ReBoot Method by assessing significance of the correlation in a simulated dataset (described below). The results are shown in Table SM1, where pairwise correlations and p-values assessed through various methods were compared. In the simulation data (Figure SM4A), b1 increased linearly while b2 remained constant in all samples; b3 also decreased linearly while b4 remained constant at a lower abundance; 16 other low-abundance microbes were also present at a constant abundance. The all microbes had low abundances and were affected strongly by the noise. The correlation and the permutation test p-values estimated from the absolute abundances were the expected values and were treated as the gold standard against which we compared our results.

Pairwise Pearson correlations calculated from the relative abundances of b1-b2 and b2-b4 pairs differed substantially from the true correlations (Table SM1) due to the effects of compositionality as expected. The p-values estimated by a simple permutation test on the relative abundance data overstated the significance of the b1-b2 and b2-b4 correlations but maintained the true significance of the b1-b3 pair. Using the permutation-renormalization null distribution (without the bootstrap distribution) improved the p-value estimates of b1-b2, but the significance level of b2-b4 correlation was still overstated. Finally, when the bootstrap distribution was introduced, the p-values of b1-b2 and b2-b4 were no longer significant, matching the expectation and the synthetic data's gold standard.

**A****B**

**C**
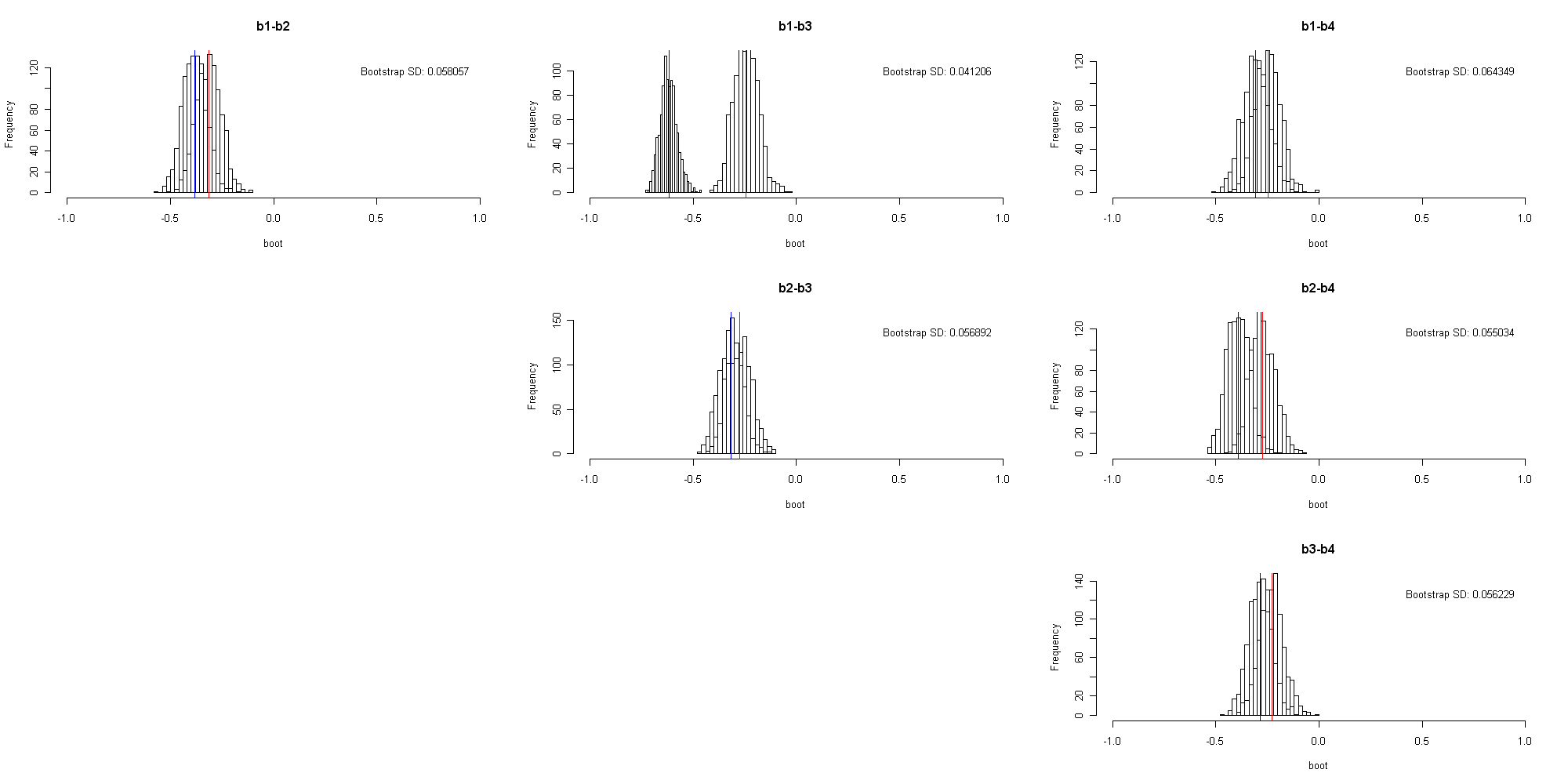


Bootstrap
 Permute+Renorm

Figure SM4 Combining a renormalized permuted null distribution with a bootstrap confidence interval adjusts the significance of associations between features appropriately according to the degree of compositionality in the data. A synthetic dataset of four simulated microbes was generated as absolute abundances (A) including a single true negative correlation between b1-b3 and uncorrelated taxa b2 and b4. (B) After normalization to relative abundances, b2 and b4 exhibit spurious correlation mitigated by (C) application of the ReBoot procedure to all pairwise comparisons. Blue and red vertical lines represent means of bootstrapped (confidence interval of true association) and permuted (null distribution of association due to compositionality) distributions, respectively. A z-test with pooled variance was used to compare the means of the two distributions, among which only the b1-b3 retains significance (see Table SM1), matching the gold standard.

Table SM1 Comparison of ReBoot p-values to a synthetic gold standard

|  | Pearson correlation | | P-value | | | |
| --- | --- | --- | --- | --- | --- | --- |
|  | Absolute abundance  (true corr.) | Relative abundance (composition) | Permutation test on the absolute abundance | Permutation test on the relative abundance | Permutation-Renormalization | Permutation-Renormalization and Bootstrap (ReBoot) |
| *b1-b2* | *-0.00043* | *-0.11471* | *0.490* | *0.039* | *0.067* | *0.114* |
| *b1-b3* | *-0.37481* | *-0.45163* | *0.000* | *0.000* | *6.69E-14* | *1.34E-13* |
| b1-b4 | 0.016205 | -0.04366 | 0.403 | 0.247 | 0.203 | 0.167 |
| b2-b3 | 0.022527 | -0.03726 | 0.359 | 0.292 | 0.264 | 0.238 |
| *b2-b4* | *-0.0603* | *-0.13541* | *0.170* | *0.013* | *0.031* | *0.082* |
| b3-b4 | 0.002101 | -0.05086 | 0.518 | 0.225 | 0.196 | 0.170 |

### Simulated data

We performed a number of simulations to demonstrate the effect of compositional measurement and assess the performance of the ReBoot method. In the simulations, absolute abundances of 10 to 100 microbes were simulated by adding normally distributed noise to a linear trend. A minimum of 10 microbes were required to capture a realistic compositional effect, while too many simulated microbes will diminish the effect. For each microbe, 250 absolute abundances (the same number as the HMP subjects) were produced as a linear function of the subject index (1-250) with random noise. The standard deviation of the noise distribution is fixed within each simulation. The true correlations between simulated microbes were determined by the correlation of the noise-free linear trends. These absolute abundances were converted to relative abundances by sample-wise normalization.

### Renormalization mitigates the compositional effect

As discussed above, an appropriate null distribution should represent the amount of correlation due to compositional measurement alone. While permutation breaks the correlation structure between pairs of microbes, it also eliminates compositional correlation. During the ReBoot procedure, since only the microbes of interest are permuted, the compositional effect is retained in the other microbes, we can thus reintroduce the compositional structure to the permuted vectors by renormalizing the data so that each sample sum is one. The correlation structure remaining in the renormalized permuted data is the correlation due to compositionality alone.

To illustrate that permutation-renormalization recovers the compositional effect, we performed a simulation (Figure SM5) in which high-abundance microbe b1 is increasing with the sample index, microbe b2 remains constant, low-abundance microbe b3 is decreasing, and the other 97 microbes are constant (and thus uncorrelated) at low abundance. The absolute abundance is normalized into relative abundance whose negative compositional (spurious) correlation between b1 and b2 is clearly visible. For each pair of microbes (e.g. b2-b3 and b1-b2 shown in Figure SM5), permutation was performed to break their correlation, then sample-wise renormalization was performed. The correlations between microbes of interest in the renormalized permuted data were the correlation structure due to compositionality alone and were visible when plotted with index ranked by abundance of the microbes of interest (e.g. b2 and b1, Figure SM5).

It is important to note that renormalization can only recapture the compositional effect in as much as one exists in the relative abundance data. Some compositional effects cannot be removed because of the information lost during the initial normalization, and in these cases ReBoot errs on the side of potential false positives, assuming that correlations greater than would be expected by chance in relative abundances were also significant in the underlying hidden absolute abundances. Again, Figure SM1 illustrates such a case where it is impossible to fully distinguish compositional, spurious correlation from the original absolute correlations.


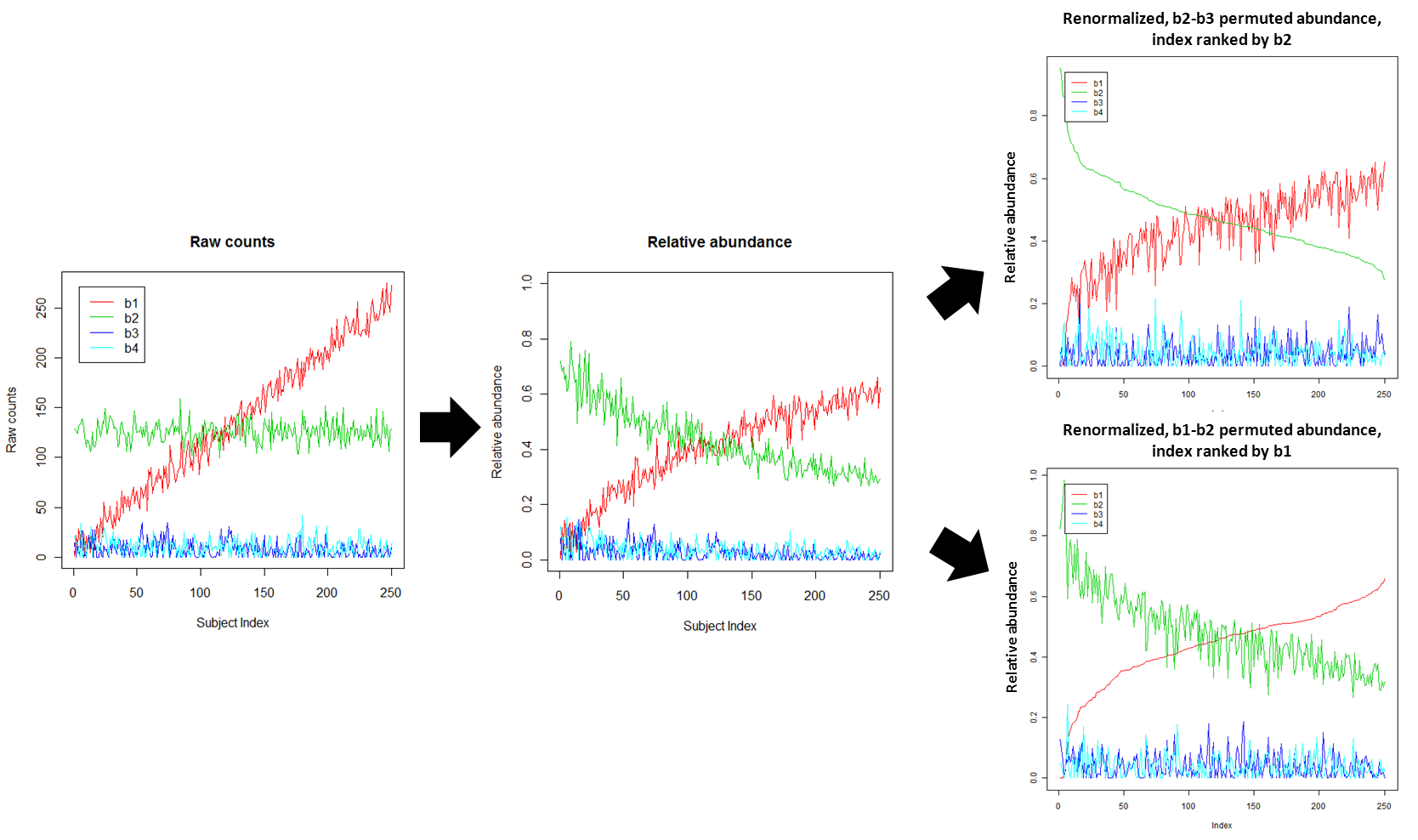


Figure SM5 Correlation due to compositionality alone is retained in the permutation-renormalization null distribution. Because permutation alone is insufficient to represent the null distribution of correlation due to compositionality, we recover the additional compositional structure through renormalization. Permutation of relative abundance breaks all correlation structure in the data, but renormalization of each column of permuted data reintroduces the compositional structure, by the degree to which it is present in the remaining non-permuted features' relative abundances. Correlations that exist in this renormalized permuted data are due to compositionality alone and can be used to construct an appropriate null distribution.

### Bootstrap scales the confidence interval according to the overall relative abundances

As discussed above, normalization reduces the information about the absolute abundance while introducing spurious correlation. It has been shown previously [[1](#_ENREF_1)] that the information about the absolute abundance can be retrieved through the variance/co-variance structure of the relative abundance. Similarly, because the noise in the data (from sequencing) is assumed to be comparable between both low and high abundance microbes, normalization affects the abundance data differently based on the noise-to-signal ratio. In particular, low absolute abundance microbes are affected more strongly by the noise, thus the variance of the relative abundance is high. The reverse is true for high abundance microbes. The variance of the relative abundance directly affects the variability of the correlations between pairs of microbes, which can be observed through the variance of the bootstrap distribution of the correlations as shown through simulations below (Figure SM6). Hence, the variability of the bootstrap distribution of the correlation can be utilized as a way to recover information about the absolute abundance of the microbes.

To demonstrate the relationship between the variability of the bootstrap distribution and the absolute abundance, we performed a series of simulations in which we monitored the shape of the bootstrap distribution of correlations between two anti-correlated vectors with increasing levels of noise (Figure SM6). In particular, for each simulation, we generated a pair of perfectly anti-correlated vectors (one is <1,2,3,…,1000> and the other <1000,999,998,…,1>). Normally distributed noise with mean zero and increasing standard deviations (Figure SM6A: 50, B: 100, C: 1000, and D: 10000) were added to the two vectors, and a bootstrap correlation distribution was generated by bootstrap sampling for each of the variance (noise) levels. Although our simulation holds the absolute abundance constant and varies the noise level, this is equivalent to holding the noise level constant and varying the absolute abundance magnitude. As shown in Figure SM6, as signal-to-noise increased, the bootstrap distribution widened, and the center shifted toward zero, which is the desired characteristic.


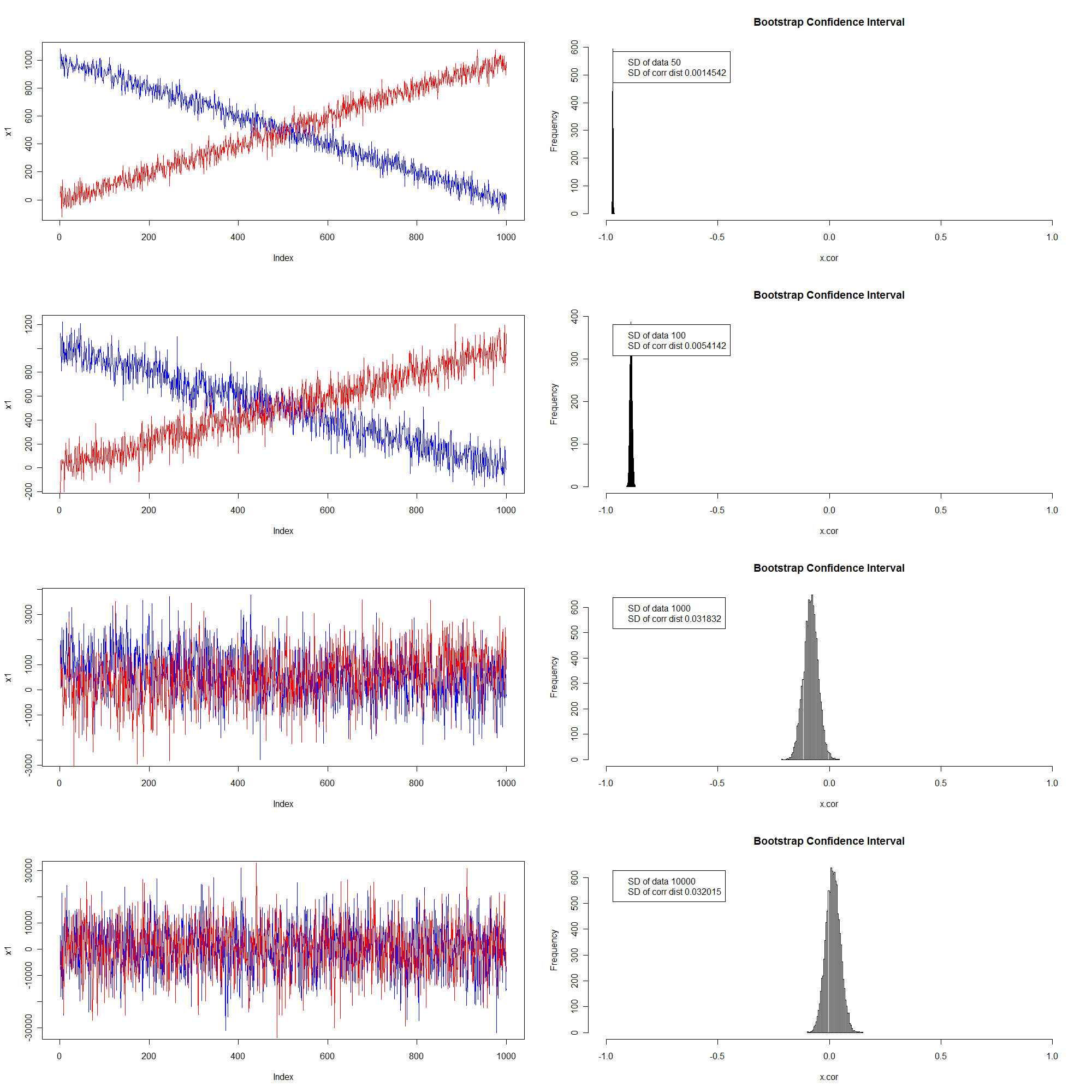


B

C

D

A

Figure SM6 The confidence interval provided by bootstrap assessment of correlations in compositional data scales by the signal-to-noise ratio, and is thus informative for excluding unreliable low-abundance signals most affected by compositionality. A series of pairs of vectors were generated with perfect negative correlations (one is <1,2,3,…,1000> and the other <1000,999,998,…,1>). Normally distributed noise with mean zero and increasing standard deviations (A) 50, B) 100, C) 1000, and D) 10000) were added, and a bootstrap correlation distribution was generated by bootstrap sampling for each of the variance (noise) levels. As signal-to-noise increased, the bootstrap distribution widened, and the center shifted toward zero.

### Filtering based on bootstrap confidence interval of R^2^ prevents over-fitting in GBLM

As an additional consideration unrelated to compositionality, we extended our application of the ReBoot procedure to GBLMs to prevent overfitting of these high-dimensional models. It has been shown that the boosting procedure for linear models can over-fit in noisy data [[2](#_ENREF_2)], and in the case of our microbial relative abundance data, low abundance microbes can have substantially noisy data and lead to over-fitting of GBLMs. In assessing significance of GBLMs, we produced bootstrap distribution of adjusted R^2^ to be compared with the null distribution, the adjustment of which provides a first step favoring simpler models with fewer parameters. However, through simulations, we observed that the bootstrapped adjusted R^2^ could in many cases remain elevated, making the bootstrap confidence interval a poor estimate of the true adjusted R^2^ and leading to potential false positives. Figure SM7 illustrates this in simulations, using data as in Figure SM5 and resulting in an over-fitted linear model b1 ~ b3 + (12 other non-informative terms). In the over-fitted model, the bootstrap distributions were not centered at the original R^2^/adjusted R^2^ values, but overinflated. Note also that the bias occurs both in R^2^ and adjusted R^2^ estimates, as the number of predictors in the model is the same between the original model and the bootstrap models and does not correct the bias discussed here. Thus, to avoid including overfit GBLMs in later analyses, we retained only GBLMs whose bootstrap 90% confidence intervals included the true (observed) R^2^ value assessed on all training data.

A
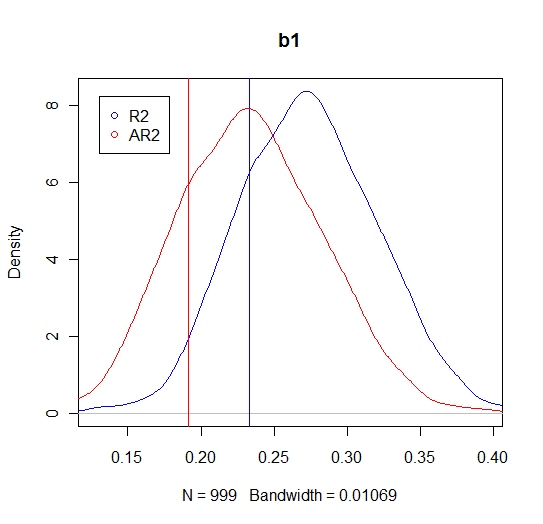
B
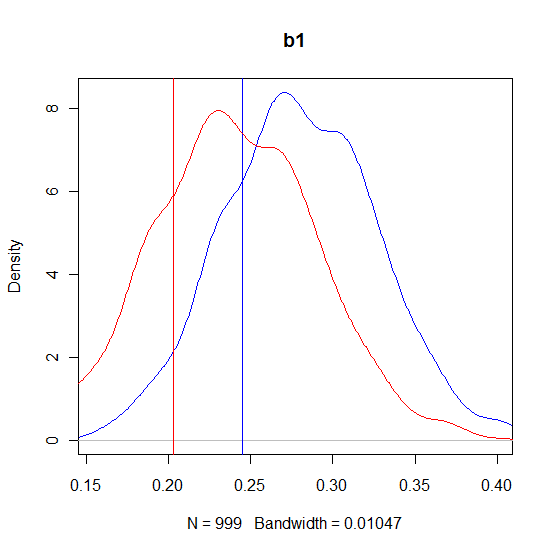


Figure SM7 The bootstrap confidence interval for a sparse linear model can be a biased estimator of adjusted R^2^. Here, we use the data of Figure SM5 in a GBLM predicting b1 relative abundance by the model: b1 ~ b3 + (12 other non-informative, low-abundance terms). R^2^ (blue) and adjusted R^2^ (red) based on the original data are shown as vertical lines and the bootstrap distributions are plotted as curves. (A) uses dense (nonzero) synthetic data and (B) demonstrates the case when the auxiliary predictors are sparse (>50% of the samples have zero abundance). The similarity between (A) and (B) suggests that the bias is independent of the sparsity of the predictors. In our application of GBLMs, to avoid including overfitting, we thus retained only models with bootstrap 90% confidence intervals including the R^2^ observed from all training data.

### Aitchinson’s log-ratio-based measure clusters with Kullback-Leibler dissimilarity

We close with a brief comment on the use of Aitchinson's proposed variance of log ratios to assess relationships between parts of compositions [[1](#_ENREF_1)]:

$$T=Var(\log\left(\frac{x}{y}\right))$$

This measure is zero if the ratio of two components is the same in all observed compositions and is increasing (without upper bound) with increasing variance of the ratio. The variance of log ratios can therefore be considered as a dissimilarity measure.

We applied the variance of log ratios to the Houston sample subset and compared the 1,000 highest- and 1,000 lowest-scoring relationships to those obtained with six other similarity and dissimilarity measures in the same data set. We found that the variance of log-ratios clustered together with other dissimilarities in terms of edge overlap, such as the Bray Curtis dissimilarity and the Euclidean distance, but shares most edges with the Kullback-Leibler dissimilarity (see Supplemental Figure 6). Because of its similarity to Kullback-Leibler, we did not include the variance of log-ratios in our analysis.

# References

1. Aitchison J (2003) A Concise Guide to Compositional Data Analysis. CDA Workshop Girona.

2. Dietterich TG (2000) An experimental comparison of three methods for constructing ensembles of decision trees: Bagging, boosting, and randomization. Machine Learning 40: 139-157.
